# Bioprospecting of Selected Species of Polypore Fungi from the Western Balkans

**DOI:** 10.3390/molecules29020314

**Published:** 2024-01-08

**Authors:** Maja Kozarski, Anita Klaus, Bojana Špirović-Trifunović, Srdjan Miletić, Vesna Lazić, Željko Žižak, Jovana Vunduk

**Affiliations:** 1 Institute for Food Technology and Biochemistry, Faculty of Agriculture, University of Belgrade, Nemanjina 6, 11080 Belgrade, Serbia; maja@agrif.bg.ac.rs (M.K.); aklaus@agrif.bg.ac.rs (A.K.); vlazic93@gmail.com (V.L.); 2 Institute for Phytomedicine, Faculty of Agriculture, University of Belgrade, Nemanjina 6, 11080 Belgrade, Serbia; spirovic@agrif.bg.ac.rs; 3 Institute of Chemistry, Technology and Metallurgy, University of Belgrade, Njegoševa 12, 11000 Belgrade, Serbia; srdjan.miletic@ihtm.bg.ac.rs; 4 Institute of Oncology and Radiology of Serbia, Paterova 14, 11000 Belgrade, Serbia; zizakz@ncrc.ac.rs; 5 Institute of General and Physical Chemistry, Studentski trg 12/V, 11158 Belgrade, Serbia

**Keywords:** biological activity, bioprospecting, *Coriolus*, *Fomitopsis*, *Ganoderma*, mushroom cultivation, oxidative damage prevention, polypore

## Abstract

Growing mushrooms means meeting challenges while aiming for sustainability and circularity. Wherever the producer is located, commercial strains are the same originating from several producers. Customized strains adapted to local conditions are urgently needed. Before introducing new species to the strain development pipeline, the chemical characterization and biological activity of wild ones need to be assessed. Accordingly, the mycoceutical potential of five polypore mushroom species from Serbia was evaluated including: secondary metabolite composition, oxidative damage prevention, anti-tyrosinase, and anti-angiotensin converting enzyme (ACE). The phenolic pattern was comparable in all samples, but the amounts of specific chemicals varied. Hydroxybenzoic acids were the primary components. All samples had varying quantities of ascorbic acid, carotene, and lycopene, and showed a pronounced inhibition of lipid peroxidation (LPx) and ability to scavenge HO^•^. Extracts were more potent tyrosinase inhibitors but unsuccessful when faced with ACE. *Fomitopsis pinicola* had the strongest anti-tumor efficacy while *Ganoderma lucidum* demonstrated strong selectivity in anti-tumor effect in comparison to normal cells. The evaluated species provided a solid foundation for commercial development while keeping local ecology in mind.

## 1. Introduction

Mushroom cultivation is speeding up in Europe driven by the widespread popularity of the vegan diet and investments in research and development of novel mushroom-based products [1,2]. Once mycophobic, the population of Western Europe is accepting mushrooms as food but also as dietary supplements as a consequence of scientific and user-based findings of fungal biological activity with few or no side effects [3]. Popular magazines, scientific journals, and specialized mushroom conferences like the International Medicinal Mushroom Conference (IMMC) are also contributing to this paradigm shift and “mushroomisation” of Europe and the Western World. However, the more they know the less satisfied with the range of mushroom products the Europeans are [4]. The biggest mushroom producers in Europe are in the Netherlands and Poland with intensive and highly industrialized facilities. They are focused on a few commercial and edible species with the highest market share, like white button, shiitake, oyster, and enoki mushrooms [5]. Such large-scale production uses standardized and certified mycelium supplied by only a few companies. Hola et al. reported that wild strains of *Agaricus bisporus* have good chemical characteristics when compared with commercial Sylvan A15 strain [6]. Moreover, small producers use the same mycelium (spawn) as the big ones, and its cost can account for up to 20% of the total production expenses while not being customized for very diverse growing conditions and equipment. This can affect the final product’s quality. When it comes to mushroom-based nutraceuticals the goal is to have high-quality raw materials rich in bioactive compounds, possibly standardized [3]. However, with the combination of small-scale diverse production and the lack of commercial spawn diversity, the quality is often compromised. Using wild strains can be a solution. Campi et al. reported that wild fruiting bodies showed higher concentrations of phenolic compounds than cultivated ones when screening bioactive compounds from medicinal mushrooms from Paraguay [7]. On the other hand, mushroom producers and mushroom-based nutraceutical producers need to adhere to and follow legislation requirements. One of them is to provide proof of the fungal strain origin (cannot be a wild strain), thus wild fruiting bodies are prohibited which severely limits options. The situation in this sector is further complicated due to the borderline nature of edible/medicinal mushrooms (legislation bodies and peers are still unfamiliar with the material) which affects the product’s legislation [8]. To mitigate these problems local wild strains need to be examined for their chemical characteristics and biological activity and evaluated for commercial development. It requires significant work and collaboration between academia and industry/entrepreneurs.

Polypore mushrooms are well-known folk remedies and part of traditional medicine in Asia and Europe. Specimens of *Fomitopsis betulina* and *Fomes fomentarius* are possibly the oldest examples of polypore mushrooms found in the bag of the approximately 5000-year-old mummy, known as the Iceman, and were probably used as multifunctional remedies [9]. In the meantime, the biological activity (anticancer, antioxidant, anti-nematode, antidiabetes, hepatoprotective, antibiofilm, and antimicrobial activity, against hyperlipidemia, anti-inflammatory and cardioprotective activity and cytotoxic toward selected cancer cell lines, etc.) of these and some other polypore species have been reported by different authors [10,11,12,13,14,15]. The most promising ones were even further tested through clinical trials and polysaccharide K, known also as krestin or PSK, from *Coriolus versicolor*, has been officially used in Japan as an adjuvant for cancer treatment [16,17]. The cultivation of polypore species is challenging as in the case of *F. betulina* (Chaga) [18,19]. If not, collecting the most promising species from the wild can be properly arranged. By knowing which species, when to collect, which growth host, as well as how to preserve, transport, store, and process, we can avoid environmental devastation while profiting from harnessing polypore power for our wellbeing.

Under the aforementioned genera, several polypore mushrooms commonly occurring in Serbia (the Western Balkan region), were selected and tested for their biological activities. Namely, five species (*Fomitopsis pinicola, F. betulina, Ganoderma applanatum, Ganoderma lucidum* and *C. versicolor*) were evaluated for their secondary metabolites, including phenolic compounds (quantity and quality), ascorbic acid, β-carotene and lycopene content, and biological activities like antioxidant potential, anti-tyrosinase activity, inhibition of ACE, and cytotoxic/antitumor activity toward selected tumor cell lines. The activities of the examined species were compared with the same species from other regions of the world and evaluated as potential sources of compounds of nutraceutical value. 

## 2. Results

### 2.1. Extraction Yields and Secondary Metabolite Content

There were statistically significant differences in the yields of all extracts, with *F. pinicola* extract being the most abundant (Table 1).

Similarly, statistically significant differences in the content of examined secondary metabolites were identified across the tested samples. The results showed that phenol concentrations were the highest among the examined compounds. TPC levels were from 60.7 to 360.3 mg GAE g^−1^ of extract DW, with *F. pinicola* having the highest level. In comparison to this study, high phenol content (278–313 mg GAE g^−1^) was found in 70% and pure ethanol extracts of *F. pinicola* from Belgrad Forest, Istanbul, and 70% ethanol extract of *F. pinicola* (264 mg GAE g^−1^) from Czech forests [20,21]. Reis et al. revealed the highest phenolics content (388 mgGAE g^−1^) in *F. pinicola* methanol extract among ten wild mushroom species collected in Bragança (Northeast Portugal) [22]. Janjusevic et al. reported 71.55 mg and 64.76 mg respectively [23]. In our study, TPC in methanol extracts of *C. versicolor* fruiting bodies obtained from Fruška Gora, a national park in Serbia, was lower (10 mg GAE g^−1^) than reported by other authors. Macakova et al. discovered substantially higher phenol content (30.7 mg GAE g^−1^) in a 70% ethanol extract of *C. versicolor* gathered in the Czech Republic’s woodlands, while Matijašević et al. reported 25.8 mg GAE g^−1^ in methanol extract of *C. versicolor* obtained from Serbia [20,24].

Vitamin C was found in high concentrations, in all of the extracts examined, ranging from 1.2 mg g^−1^ (*G. applanatum*) to 3.1 mg g^−1^ (*C. versicolor*) (Table 1). Besides being at the lowest concentration in *G. applanatum* extract, it was significantly higher than the vitamin C content of methanol extract (0.016 mg g^−1^) of *G. applanatum* fruiting bodies collected from Gaurishankar Conservation Area, Central Nepal [25]. Reis et al. reported 1.08 mg g^−1^ of vitamin C in methanol extract of *F. pinicola* collected in Bragança (Northeast Portugal. It was almost 50% lower than the vitamin C content measured in our study for the *F. pinicola* extract, Table 1 [22]. Moreover, the vitamin C content of investigated polypore extracts was higher than its content in some fruits e.g., apples, pears, and plums 3–5 mg 100 g^−1^ [26].

There were statistically significant differences in the quantity of carotenoids, lycopene, and its metabolite β-carotene, among the studied extracts (Table 1). In the *F. pinicola* extract β-carotene and lycopene were found to be at the highest concentrations among the tested extracts (541.5 μg g^−1^ and 265.7 μg g^−1^, Table 1. Reis et al. reported a significantly lower concentration of β-carotene (22 μg g^−1^) in the methanol extract of *F. pinicola*, while lycopene was not detected [22].

#### Phenolic Profile of the Methanol Extracts

Table 2 shows the polyphenol composition of methanol extracts of five lignicolous mushroom species. The extracts included nine of the total thirteen analyzed phenolic acids and their derivatives. The most abundant, according to the results, were hydroxybenzoic acids (HBA). Gallic acid was the primary phenolic element in *F. pinicola* extract, while *C. versicolor* extract had the least amount, nearly 99% less than *F. pinicola* extract (Table 2). Furthermore, Onar et al. discovered that gallic acid was one of the primary constituents (5.5333 ± 0.0181 ppm) of *F. pinicola* ethanol extract determined by HPLC [21]. The most abundant phenolic acid in *F. betulina* extract was p-hydroxybenzoic acid (94.27 μg g^−1^ DW), while it was the least abundant in *C. versicolor* extract (0.24 μg g^−1^ DW). p-Hydroxybenzoic acid and its esters, sometimes known as parabens, commonly occur in bound form and are typically a component of a complex structure in mushrooms such as lignins and hydrolyzable tannins [23]. Protocatechuic acid, a p-hydroxybenzoic acid derivative, was found in all of the extracts tested. The minimum and maximum protocatechuic acid values in *G. lucidum* and *F. betulina* were 5.26 μg g^−1^ and 29.94 μg g^−1^, respectively. Ellagic acid was detected in methanol extracts of *F. pinicola* and *G. applanatum* (31.23 μg g^−1^ and 22.03 μg g^−1^). Ellagic acid is derived from ellagitannins, and for comparison, it is widely found in a variety of plants and fruits (berries and walnuts) [27]. Vanillin was found in extracts of *G. lucidum*, *F. pinicola*, and *G. apllanatum* at concentrations ranging from 0.81 to 1.81 μg g^−1^. Vanillic acid, an oxidized form of vanillin, was found in extracts of *F. betulina* and *G. applanatum*. Vanillic acid is widely used as a flavoring agent in various food products and drinks, pharmaceutical, and cosmetics industries [28]. Syringic acid was detected in extracts of *F. pinicola*, *F. betulina*, and *G. applanatum* in the range of 3.07–9.34 μg g^−1^. Among the hydroxycinnamic acids (HCA), chlorogenic acid was found in low amounts in extracts of *F. betulina*, *F. pinicola*, *G. applanatum*, and *G. lucidum* (Table 2). Caffeic acid was exclusively found in *G. lucidum*. It can be found in foods like coffee, wine, and tea, as well as popular medications like propolis [29]. p-Coumaric acid was detected in *F. betulina*, *F. pinicola*, and *G. lucidum* in concentrations ranging from 0.49 to 18.24 μg g^−1^, respectively. This acid was not present in the extracts of *F. pinicola* and *C. versicolor*. On the contrary, Janjusevic et al. (2017) discovered p-coumaric acid (1.28 μg g^−1^) in *C. versicolor* methanol extract [23]. Furthermore, Nowacka et al. reported p-coumaric acid (0.56 μg g^−1^ DW) in an ethanol extract of *F. pinicola* gathered in the forests of Lublin Province, Poland [30]. All similarities and discrepancies in the presence and concentration of phenolic acids found in this study and the literature can be explained by the variety of growing circumstances, extraction methods, and growing environments, as well as stressors like UV radiation, virus, and parasite infection, damaging air pollution, and exposure to severe temperatures/altitude above sea level [31].

In this investigation, the flavonoid profile was compared to five standards: quercetin, rutin, epicatechin, kaempferol, and resveratrol (Supplementary Materials Figures S1 and S2). Different mushroom species have been found to contain quercetin, rutin, catechin, and kaempferol [32]. In contrast to these findings, LC-MS/MS analysis of flavonoid concentrations in methanol extracts was below the limit of the detection (LOD) of 0.005 µg mL^−1^. Furthermore, the presence of stilbenes, such as resveratrol, was also in the LOD value. In comparison, Janjušević et al. reported by HPL-MS/MS that quercetin (29.30 μg g^−1^) and catechin (21.90 μg g^−1^) were the primary flavonoid constituents of *C. versicolor* methanol extract from Fruška gora, Vojvodina, Northern Serbia [21]. Moreover, Erbiai et al. confirmed that quercetin had the highest content (947.2 μg g^−1^ dw) at the *G. lucidum* methanol extract from the Biological and Ecological Interest Site (SIBE) of Koudiat Taifour in northwestern Morocco [33]. In this extract, the presence of rutin, narigenin, apigenin, and kaempferol was also detected [33]. However, Onar et al. reported that the content of epicatechin, catechin hydrate, and quercetin dihydrate determined by HPLC was ≤1 ppm in *F. pinicola* ethanol extract from Belgrad Forest, Istanbul [21].

### 2.2. Antioxidant Potential

**Figure 1:**
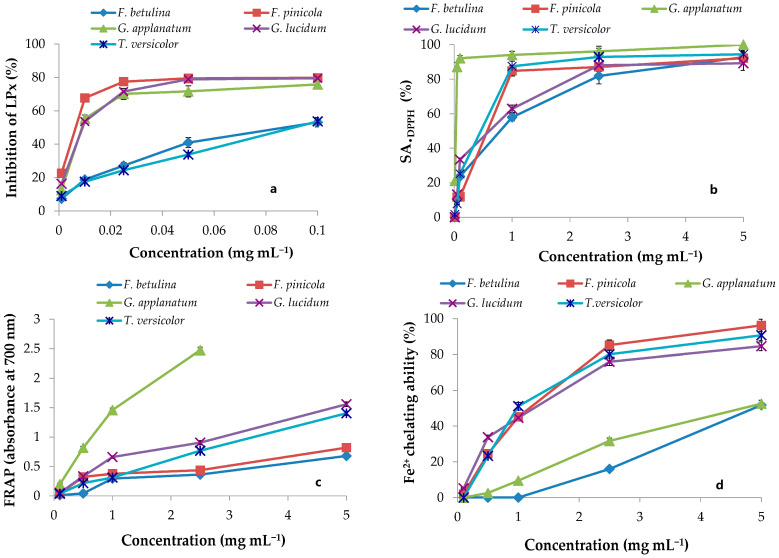
Antioxidant activity of polypore mushroom methanol extracts; inhibition of LPx evaluated in the linoleic acid model system (**a**), scavenging ability on DPPH radicals (SA_•DPPH_) (**b**), ferric-reducing antioxidant power (FRAP) (**c**), and Fe^2+^ chelating ability (**d**). Each value is expressed as mean ± SEM (*n* = 3).

**Figure 2:**
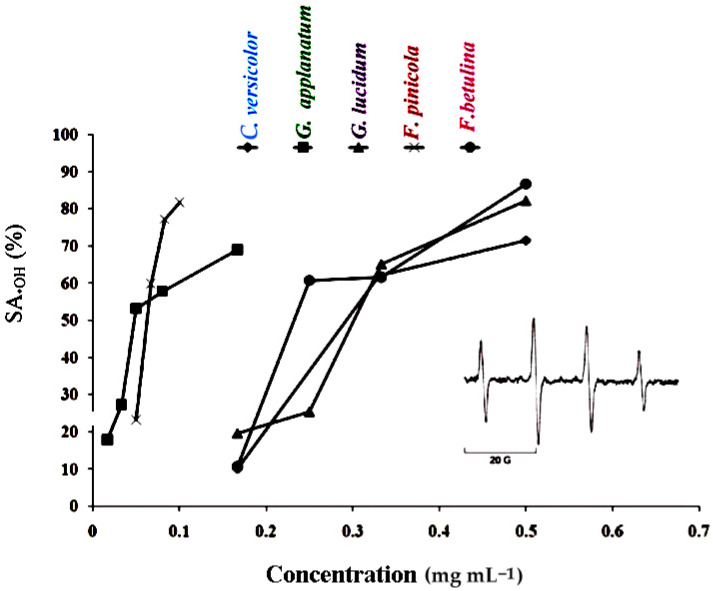
•OH scavenging activity (SA_•OH_) of polypore mushroom methanol extracts. The inserted figure represents the ESR spectrum of DMPO-OH spin adducts (error bars are too small and not visible).

Five complementary experiments were performed in the current investigation to gain a comprehensive understanding of the antioxidant activities of the methanolic extracts of the five polypore mushroom species, and the findings are shown in Figure 1 and Figure 2, as well as in Table 3.

#### 2.2.1. Scavenging Ability

The •DPPH and •OH tests were used to measure the scavenging capacity. The •DPPH assay can be used to determine the antioxidant capacity of complex samples in which HAT (hydrogen atom transfer), ET (electron transfer), and proton-coupled electron transfer (PCET) mechanisms may play different roles in different proportions depending on the corresponding reaction conditions (such as pH and solvent) [34]. Each extract’s percentage of •DPPH inhibition was found to be high (Figure 1). At 5 mg mL^−1^, the greatest percentages of inhibition were 92.18, 92.77, 100, 89.24, and 94.44 in *F. betulina*, *F. pinicola*, *G. applanatum*, *G. lucidum*, and *C. versicolor* methanol extracts, respectively (Figure 1). Among the extracts tested, *G. applanatum* had the highest •DPPH scavenging capacity (IC_50_) value of 48 μg mL^−1^ (Table 1) when compared to the conventional additions of ascorbic acid (IC_50_ 56 μg mL^−1^) and alpha-tocopherol acetate (39 μg mL^−1^). In the study of Karaman et al., eight fungal species originating from Serbia were evaluated for •DPPH scavenging capacity [35]. *G. applanatum* methanol extract was also the most promising with 82.80% of •DPPH inhibition at the 12.5 μg mL^−1^. Onar et al. reported IC50 values of the 70% ethanol extract of *F. pinicola* to be 0.30 ± 0.005 mg mL^−1^ and it was comparable to the IC_50_ value of *F. pinicola* methanol extract in our study (Table 1) [21]. In contrast, Reis et al. confirmed that the IC_50_ value in •DPPH scavenging activity of *F. pinicola* methanol extract was moderately lower in comparison to the IC_50_ value of *F. pinicola* extract in this study [22]. •OH scavenging activity of the examined methanol extracts are presented in Figure 2 and Table 3. •OH may react with organic compounds in two ways: hydrogen atom (or electron) abstraction and (slightly faster) addition to double bonds [36]. This assay reveals not only a scavenger’s ability to react with •OH but also its ability to form complexes with Fe^2+^, influencing its redox behavior in the Fenton reaction. *F. pinicola* extract was the most effective antioxidant in scavenging •OH, exhibiting 83% activity as early as 0.01 mg mL^−1^. For comparison, at 0.5 mg mL^−1^, *F. betulina* and *G. lucidum* extracts had the same impact (SA_•OH_: 85% and 82%, respectively). Karaman et al. confirmed that methanol extracts of *G. applanatum* showed the highest scavenging effect on •OH (68.47%) at 0.4 mg mL^−1^ [35]. Contrary to this finding, methanol extracts of *G. applanatum* from the current study showed the same scavenging ability on •OH at a much lower concentration, 0.18 mg mL^−1^ (Figure 2).

#### 2.2.2. LPx Inhibition

Given the fact that linoleic acid is among the most prevalent polyunsaturated fatty acids (PUFA) in cell membranes [37], in this research, the total antioxidant activity in the protection of LPx in the linoleic acid model system was studied. The mean EC_50_ values for *F. pinicola* and *G. applanatum* suggested that they were the most powerful antioxidants in the protection of linoleic acid peroxidation, with values of 12 and 18 μg mL^−1^, respectively (Table 3). In addition, *F. betulina*, *G. lucidum,* and *C. versicolor* extracts were effective linoleic acid oxidation inhibitors. They had lower EC_50_ values (Table 3) than ascorbic acid (1.65 ± 0.08 mg mL^−1^), a typical food ingredient utilized at mg levels. One of the most effective antioxidants extensively employed in industry is alpha-tocopherol acetate, with an EC_50_ value of 36 μg mL^−1^. Mushroom extracts are effective inhibitors of LPx [35,38]. However, it is important to emphasize that in the literature, the most common method used for the assessment of the LPx inhibition is the thiobarbituric acid reactive substances (TBARS) method. Due to its complexity (long incubation period and specific equipment), the conjugated diene method is rarely used in the examination of the antioxidant potential of mushroom extracts. Thus, in this study comparison of the LPx inhibition potential, which was measured in the model system of linoleic acid, was compared with the most comparable method of inhibition, β-carotene bleaching in the linoleic acid system. According to this method, Reis et al. showed that EC_50_ values of *F. pinicola* and *F. betulina* were 0.10 and 1.97 mg mL^−1^ [24]. It is in contrast to the results obtained in this study, where *F. pinicola* and *F. betulina* showed excellent and much higher potential for the LPX inhibition (Table 3).

#### 2.2.3. FRAP Assay

Reductones can exert an antioxidant effect by interrupting the free radical chain reaction by donating a hydrogen atom [39]. In the FRAP experiment, newly generated electron donors provided by antioxidants can decrease Fe^3+^ to Fe^2+^. This reducing capacity of substances could be used to predict possible antioxidant capabilities, and increased absorbance indicates a rise in reducing power [40]. Concentration-dependent, the reducing capabilities of mushroom methanol extracts increased with increasing concentration (Figure 2). *G. applanatum* extract had the highest increase in absorbance (2.48 at 2.5 mg mL^−1^) and the lowest EC_50_ value (0.30 mg mL^−1^) of the five extracts (Figure 2 and Table 3). In the FRAP assay, ascorbic acid had a lower EC_50_ value (0.056 ± 0.007 mg mL^−1^) and stronger reducing power than the examined extracts. In comparison to this study, Sułkowska-Ziaja et al. reported that methanol extract from the *G. applanatum* mycelium showed a reducing capacity of 8.10 mg TE g^−1^ [41], while Reis et al. reported EC_50_ values of the reducing potential of methanol extracts of *F. pinicola* and *F. betulina* of 0.06 mg mL^−1^ and 1.64 mg mL^−1^, respectively [22].

#### 2.2.4. Capability to Chelate Fe^2+^

When testing chelating ability on ferrous ions, methanol extracts in this study showed different patterns of chelating ability at 0.01–5 mg mL^−1^ (Figure 2 and Table 3). *F. pinicola* and *C. versicolor* extracts displayed the best Fe^2+^ chelating activity among the extracts at 5 mg mL^−1^, 96.3%, and 90.7%, respectively (Figure 2). These samples also had the lowest EC_50_ values (0.99 and 1.02 mg mL^−1^) (Table 3). Results expressed as Fe^2+^ equivalents were for *F. pinicola* 0.20 mM Fe^2+^ g^−1^ and *C. versicolor* 0.19 mM Fe^2+^ g^−1^ of extract dw, respectively. Ascorbic acid had an EC_50_ value of 0.49 mg mL^−1^ or 0.41 mM Fe^2+^ g^−1^ dw. At 0.1–5 mg mL^−1^, the chelating effect of the synthetic metal chelator EDTA was between 91% and 100% while citric acid was not an effective chelating agent. At 5 mg mL^−1^, its chelating ability was 3.4%. In the study by Mau et al., *C. versicolor* methanol extract showed chelating effects on Fe^2+^ of 13.2% at 2.4 mg mL^−1^ [42]. It was significantly lower than the chelating ability of the *C. versicolor* methanol extract from this study for the same concentration (78.9% at the 2.4 mg mL^−1^). In opposition to this finding, Mau et al. showed that *G. lucidum* methanol extract expressed strong chelating effects on Fe^2+^, 67.7% at the 2.4 mg mL^−1^ [42]. It was comparable with the potential of the *G. lucidum* methanol extract analyzed in this study (75.1% at the 2.4 mg mL^−1^).

#### 2.2.5. Correlation between Antioxidant Activity and Components of Methanol Extracts

**Figure 3:**
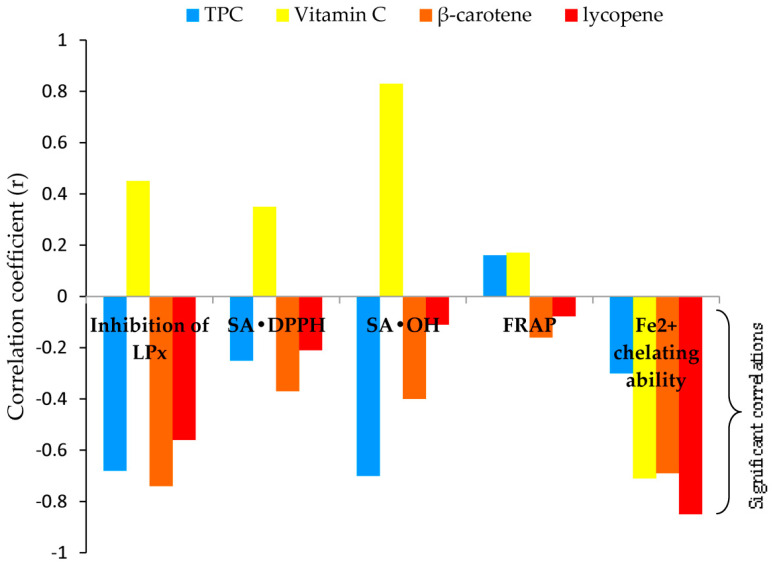
Relationship between EC_50_ values in antioxidant activities and analyzed secondary metabolite content.

To better understand the mechanism of antioxidant action, the correlation coefficients (*r*) among results obtained with antioxidant assays, and antioxidant assays with bioactive components of methanol mushroom extracts were determined (Figure 3, Table S1).

Correlations among results obtained with antioxidant assays were positively very strong for SA_•DPPH_ and FRAP (*r* = 0.85); positively strong for inhibition of LPx and SA_•DPPH_ (*r* = 0.68), inhibition of LPx and SA_•OH_ (*r* = 0.76), and SA_•DPPH_ and SA_•OH_ (*r* = 0.62); moderate, positive correlation was observed between inhibition of LPx and FRAP (*r* = 0.57). There was no significant correlation between the chelating ability of Fe^2+^ and other assays (detailed information is given in Supplementary Materials, Table S1).

As a confirmation, the correlations between antioxidant assays and the content of bioactive components inferred, Figure 3: 1. TPC—strong and negative correlation with SA_•OH_ and inhibition of LPx (*r* = −0.70 and *r* = −0.68), and weak with chelating ability of Fe^2+^ and SA_•DPPH_ (*r* = −0.30 and *r* = −0.25); 2. Vitamin C—strong and negative correlation with chelating ability of Fe^2+^ (*r* = −0.71); 3. β-carotene—strong correlation with inhibition of LPx and chelating ability of Fe^2+^ (*r* = −0.74 and *r* = −0.69), moderate with SA_•OH_ (*r* = −0.40), and weak with SA_•DPPH_ (*r* = −0.37); 4. Lycopene—very strong correlation with chelating ability of Fe^2+^ (*r* = −0.85), moderate with inhibition of LPx (*r* = −0.56), and weak with SA_•DPPH_ (*r* = −0.21).

### 2.3. Enzyme Inhibition

#### 2.3.1. Tyrosinase Inhibitory Potential

The inhibitory potential for tyrosinase exhibited by methanol extracts is presented in Table 3; all samples had the ability to inhibit this enzyme. *F. pinicola* extract displayed the strongest inhibitory activity with the lowest IC_50_ values (0.10 mg mL^−1^) and exhibited a slightly weaker potential when compared with kojic acid (0.079 ± 0.009 mg mL^−1^) commonly used as a standard inhibitor of tyrosinase. It is important to emphasize that in the literature, the IC_50_ values for tyrosinase inhibitors are commonly incomparable due to the variations in assay conditions, including different substrate concentrations, varied incubation times, and different batches of commercial tyrosinases [43]. Thus, most studies utilized a well-known tyrosinase inhibitor, such as kojic acid as a positive control in an effort to normalize the inhibitory activities of their inhibitors.

**Figure 4:**
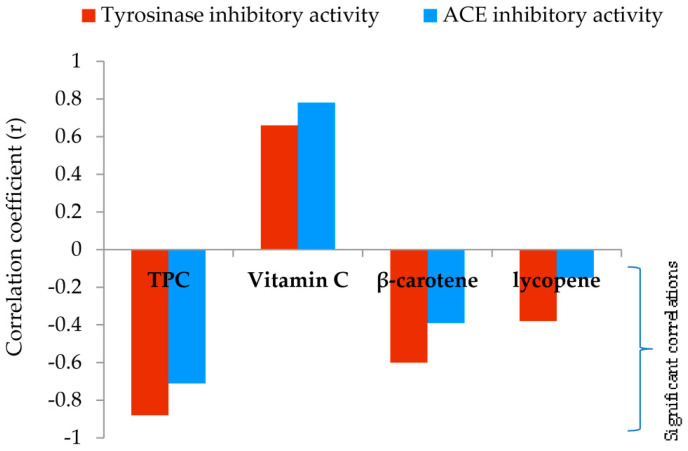
Relationship between IC_50_ values in enzyme inhibition and analyzed secondary metabolite content.

In addition, a significant and very strong negative correlation (*r* = −0.88) between the TPC of methanol extracts and tyrosinase inhibition was reported (Figure 4, detailed information is given in Supplementary Materials—Table S2). Furthermore, a strong correlation (*r* = −0.60) was observed between the total content of β-carotene and tyrosinase inhibition (Figure 4).

#### 2.3.2. Anti-ACE Potential

As shown in Table 3 the results revealed varying levels of ACE inhibitory potential among tested samples. *F. pinicola* exhibited the highest ACE inhibitory activity with an IC_50_ value of 0.65 mg mL^−1^. *G. lucidum*, *F. betulina*, and *G. applanatum* also demonstrated notable inhibitory activity, registering values of 1.28, 1.01, and 0.89 mg/mL respectively. On the other hand, *C. versicolor* exhibited the lowest ACE inhibition, with an IC_50_ value of 1.96 mg/mL. To normalize the inhibitory activities of the investigated extracts, a well-known ACE inhibitor such as captopril was selected. Captopril, the first oral ACE inhibitor commercially applied for the treatment of hypertension, showed an IC_50_ value of 0.006 ± 0.000 mg mL^−1^. TPC had the most effect on ACE inhibition (Figure 3, Table S2). Regression analysis revealed a strong correlation (*r* = −0.71) between the TPC of methanol extracts and ACE inhibition.

### 2.4. In Vitro Cytotoxic Activity

The decrease in the survival of target cells induced by methanol extracts is presented in Table 4. In general, all extracts exerted statistically significant differences in the cytotoxic activity against the target malignant cell lines after 72 h of incubation. *F. pinicola* extract possessed the highest activity with IC_50_ values in the range of 23–29 μg mL^−1^ against the malignant cell lines. *C. versicolor* extract displayed less pronounced cytotoxicity, with IC_50_ values from 220–251 μg mL^−1^. Itharat et al. indicated that the criteria of cytotoxicity for crude plant extracts, as established by the American National Cancer Institute (NCI), is an IC_50_ < 30 μg mL^−1^ in the preliminary assay [44]. According to NCI criteria, the methanol extract of *F. pinicola* displayed moderate cytotoxicity against the malignant cell lines as well as normal BEAS-2B and MRC-5 cells. However, according to the same criteria the other extracts had no cytotoxic activity. Their IC_50_ was more than 30 µg mL^−1^, Table 4. IC_50_ value of cisplatin, which served as a positive control, in the cytotoxicity testing against K562 and HeLa cells was <15.6 μg mL^–1^, and against MDA-MB-453 was 19.2 μg mL^–1^, respectively.

#### Selectivity Coefficient in the Cytotoxicity against Tumor Cells

**Figure 5:**
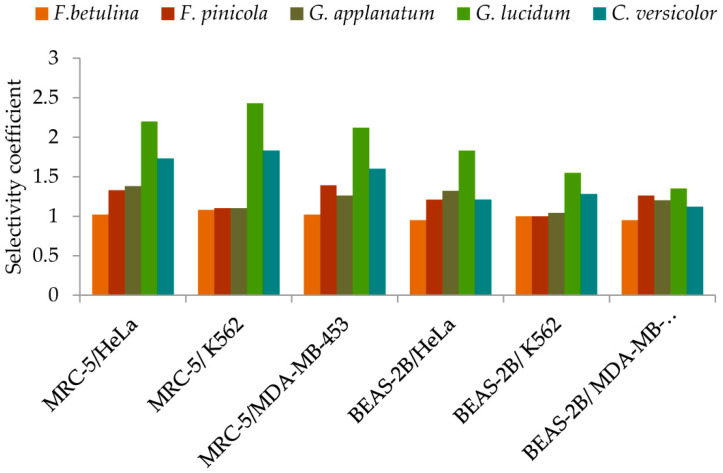
Selectivity of methanol extracts in cytotoxicity against tumor cells.

Considering the possible effects of antitumor drugs on normal cells, which are a normal part of the tumor microenvironment, the activities of the investigated extracts were evaluated against normal MRC-5 and BEAS-2B cells (Figure 5, detailed information is given in Supplementary Materials—Table S3). Extracts exhibited different cytotoxic effects against normal cells. Generally, MRC-5, normal lung fibroblasts, were less sensitive to the tested extracts than BEAS-2B, bronchial epithelial cells (Figure 5). MRC-5 cells contribute to the formation of connective tissue and secrete collagen, elastin, and other extracellular matrix proteins that help maintain the structural framework of tissues. Likewise, it was observed that *G. lucidum* extract exhibited significantly higher selectivity between all tested malignant and normal cells compared to other extracts (Figure 5, Table S3). Contrary, no selectivity was observed for *F. betulina* extract. *F. pinicola* and *G. apllanatum* extract had no selectivity in cytotoxic action between normal and K562 cells (Figure 5, Table S3).

## 3. Discussion

Three different types/classes of secondary metabolites of polypore mushroom methanol extracts were examined for their contents: polyphenols, vitamin C, and the carotenoid lycopene and its metabolite β-carotene. These secondary metabolites have a crucial role as antioxidants in keeping cells in a reduced environment, shielding them from oxidative damage, and ensuring correct cell function [25,26]. Moreover, as signaling pathway modulators, they can influence cell regulation e.g., enzyme activity, cytokine generation, and gene expression [32,45,46]. With regard to these activities, selected polypore extracts rich in secondary metabolites were assessed for their antioxidant properties, potential for enzyme inhibition, and cytotoxicity against tumor cell lines. Selection of the most potent polypore species (often occurring in Serbia, the Western Balkan region) provides guidelines for its commercialization and potential application as a food additive and nutraceutical formulation.

### 3.1. Polyphenols

In recent years polyphenol-rich mushroom extracts have been commercially available and according to their widespread structural diversity have been increasingly popular in the healthcare industry and cosmeceutical formulations [23]. Concerning possible dietary application, it is crucial to note that polyphenol content varies depending on the location and environmental conditions of growth as well as with content of secondary metabolites with protective effects against radiation, mechanical damage, and microbial infection [47,48,49]. Besides, mushrooms possess the ability to absorb polyphenols from the substrate on which they grow or from neighboring plants [25]. This could explain the differences in content and composition across investigated polypore species gathered from various locations, Table 2 and Table 3. At the same time, this emphasizes the significance of identifying the most potent species and developing appropriate growing techniques as well as spawn suitable for the local conditions.

The most abundant phenolic compounds, according to the results, were phenolic acids, Table 3. Phenolic acids might be used in direct interactions with radical species (RS) as enhancers of cell antioxidant defenses and to reestablish redox balance through primary (chain breaking) and secondary mechanisms, e.g., chelating/deactivation of metals, inhibition or breakdown of lipid hydroperoxides [36,48]. The importance of these antioxidant compounds is related to both their concentration and reactivity [24]. In this regard, different RS can react via different primary and secondary mechanisms in different physiological media. Five assays based on different stages of the oxidation process and on different mechanisms were performed in the current investigation to gain a comprehensive understanding of the antioxidant activities of the methanolic extracts of the five polypore mushrooms, Table 3. FRAP measures the ability to reduce metals while •OH and •DPPH assays measure a sample’s free radical scavenging capacity [36]. In FRAP, a SET reaction mechanism occurs. With •DPPH and •OH, both HAT and SET reactions as well as proton-coupled electron transfer (PCET) occur. Besides, •OH can act via radical adduct formation (RAF) to double bonds [36]. Chelating ability measures the potential of investigated metabolites to bind ferrous ions and form complex structures. Inhibition of LPx includes all chain-breaking mechanisms that can protect lipids from oxidative deterioration (HAT, SET, PCET, RAF) [36]. Results of regression analysis indicated that TPC exerted the most significant impact on the ability of analyzed samples to primarily act as •OH scavengers and to reduce LPx in the linoleic model system (Figure 3, Table S1) This could be explained by the high ability of the phenolic acids to donate hydrogen and electron and to react to several mechanisms of the scavenging actions of free radicals, e.g., HAT, ET, PCET as well as RAF [36,38]. In addition, most of the identified phenolic acids in the present study were known for their high antioxidant activity [50]. It is important to mention that as antioxidants, phenolic acids have also a wide range of industrial applications [49,51]. Gallic acid is employed as a chelating agent and preservative in food and drinks and in the skin and leather industries [52]. p-Hydroxybenzoic acid is also utilized as a preservative in a variety of medications, cosmetics, pharmaceuticals, foods, and beverages [52]. Moreover, in ex vivo studies, Salau et al. confirmed that vanillin and vanillic acid protect against oxidative brain damage by increasing ATPase activity while inhibiting cholinergic enzymatic activity [53].

Besides being well-known antioxidants, polyphenols may influence multiple immunomodulatory processes involved in inflammatory response and carcinogenesis [31,48,52]. Each type of polyphenol e.g., phenolic acids, can target and bind to one or more proteins and/or receptors on cells thus triggering intracellular signaling pathways that ultimately regulate the host response, from central metabolism to signaling events [48,49,54]. This binding, mainly non-covalent, includes hydrophobic and van der Waals interactions, ionic interactions, and H bridge/bonding, and it is always reversible [54]. In this manner, cytotoxicity against tumor cells was observed in this study, Table 4. For example, the presence of methoxy groups in the aromatic ring at positions 3 and 5 is ascribed to syringic acid’s medicinal action. It has the ability to influence enzyme activity, protein dynamics, and the activity of many transcription factors implicated in inflammation, cancer, and angiogenesis [27]. Furthermore, it was reported that kinases are among the major targets of polyphenols [49]. Protein kinases are important therapeutic targets in cancer because of their critical role in signaling mechanisms that drive malignant cell characteristics [55]. They have the potential to bind to ATP-binding sites thereby modulating the action of mitogen-activated protein kinase (MAPK), phosphoinositide 3-kinase (PI3K), Akt/protein kinase B (Akt/PKB), tyrosine kinases, and protein kinase C (PKC) pathways. Inhibiting or stimulating these pathways influences phosphorylation events and modulation of gene expression [49,55]. The fact that various polyphenols interact with cyclin-dependent kinases (CDKs) highlights their potential control of cell cycle events, including cell proliferation and cancer cell development [49]. Moreover, it has been observed that compounds with gallate groups, present in mushrooms, can target/upregulate different cellular receptors e.g., 67 kDa laminin receptor (67LR) and zeta chain-associated 70 kDa protein (ZAP-70) which are abnormally expressed in multiple cancers [48]. Additionally, it has been shown to regulate multiple crucial cellular signaling pathways, including those mediated by epidermal growth factor receptor (EGFR), janus kinase/signal transducers and activators of transcription (JAK/STAT), MAPKs, nuclear factor kappa-light-chain-enhancer of activated B cells (NF-κB), phosphoinositide 3 kinase/Akt/mammalian target of rapamycin pathway (PI3K-AKT-mTOR), among others [56]. Deregulation of the abovementioned pathways is involved in the pathophysiology of cancer. In our study, *F. pinicola* extract with the highest TPC and gallic acid content (Table 1, Table 2 and Table 4) possessed the best anti-tumor activity against malignant cell lines (K562, HeLa, and MDA-MB-453). However, it is important to mention that according to NCI criteria, the other extracts had no significant cytotoxic activity against malignant cells in the preliminary assay (Table 4). Their TPC was from 3.5 times to almost 13 times lower than in *F. pinicola* extract (Table 1).

Due to the ability of phenols to react with proteins, in this study the potential inhibition of polypore methanol extracts against tyrosinase and ACE was investigated. Tyrosinase is widely present in fruits, mushrooms, and vegetables [51], and is responsible for enzymatic browning reactions in damaged foodstuff during post-harvest handling and processing. Accordingly, its inhibitors act as anti-browning compounds and have an important role in the control of food quality. Also, in the cosmetic industry, inhibitors of tyrosinase have important applications as skin-lightening agents [57]. ACE inhibition is a big challenge today. ACE inhibitors are used for the prevention of hypertension, cardiovascular diseases, and congestive heart failure. Hypertension stands as the primary cause of premature death worldwide [57]. *F. pinicola* extract displayed the strongest inhibitory activity almost comparable with kojic acid (0.079 mg mL^−1^) commonly used as a standard inhibitor of tyrosinase, Table 3. A very strong and significant correlation between TPC and tyrosinase inhibition was observed (Figure 4, detailed information is given in Supplementary Materials—Table S2). Gallic acid was found to be the main polyphenol ingredient of *F. pinicola* extract. It has already been confirmed that this acid displays tyrosinase inhibitory activity [58]. Gallic acid can act as a substrate of tyrosinase and also can reduce dopaquinone back to levodopa (L-DOPA) through redox cycling, similar to ascorbic acid [45]. In addition, the anti-tyrosinase activity exhibited by investigated methanol extracts could be also attributed to the presence of other phenolic acids like protocatechuic, p-hydroxybenzoic, chlorogenic, vanillic, p-coumaric, and caffeic acid [59]. An example is a confirmation that protocatechuic acid may significantly suppress the melanin content and cellular tyrosinase activity in α-melanocyte stimulating hormone-stimulated mouse melanoma cells [59]. Furthermore, TPC had the highest influence on ACE inhibition (Figure 4, Table S2). However, *F. pinicola* extract which was the best in ACE inhibition expressed almost 100 times weaker inhibition potential compared to the captopril (0.006 mg mL^−1^), often applied for the treatment of hypertension. Contrary to this study, our previous investigation of the ACE inhibition potential of the birch polypore *F. betulina* (formerly known as *Piptoporus betulinus*) hot alkali extract from the national park Divčibare, Serbia showed promising results with the EC_50_ value of 0.06 ± 0.0 mg mL^−1^ [9]. The TPC of *F. betulina* alkali extract (40.1 mg GAE g^−1^) was comparable to the TPC values of the *F. betulina* methanol extract analyzed in this study (32.6 mg GAE g^−1^). However, ACE inhibition potential of the methanol extract (Table 3) was much lower than *F. betulina* alkali extract. Many studies have demonstrated that mushroom-derived compounds can be considered as a functional food, with an antihypertensive effect, expressed by inhibition of ACE [60,61]. Peptides were identified as ACE inhibitors in most of the studies [60,61,62]. Unlike the investigated methanol extracts presence of proteins/peptides in the *F. betulina* alkali extract was confirmed (40 mg g^−1^ dw) [9]. Thus, this may indicate that mushroom peptides have a primary role as potential ACE inhibitors in relation to their polyphenols ingredients.

The presence of flavonoids was not observed among methanol extracts. Various findings and conclusions have been published by scientists on the metabolic entry site for flavonoid biosynthesis in mushrooms and the presence or absence of the enzymes chalcone synthase (CHS) and chalcone isomerase (CHI), as well as other enzymes involved in flavonoid biosynthesis [32]. A couple of studies suggest that the abundance of flavonoids in mushrooms is related to these organisms’ ability to absorb various nutrients and chemicals from the substrate on which they grow or from surrounding plants by spreading their hyphae or creating mycorrhizae [63]. In contrast to this result, Mohanta [64] reports, for example, the presence of chalcone-flavanone isomerase (CHFI), which is related to flavonoid production in the lignicolous mushroom *Lentinula edodes* in his newest report on genome-wide research throughout the genomic sequences of the fungal kingdom [64]. The author did note, however, that the existence of genes involved in the flavonoid production pathway is not uniform in fungi. Shao et al. investigated secondary metabolites in six *Sanghuangporus sanghuang* strains which are employed for large-scale commercial manufacturing, and indicated the presence of seven flavonoid biosynthesis-related genes [63]. Furthermore, it was discovered that *S. sanghuang* produces more flavonoids in mycelial stages than in fruiting body stages [65].

### 3.2. Lycopene and β-Carotene

Lycopene and β-carotene contents in the investigated polypore extracts were in a wide range of concentrations, from several tens to several hundred μg g^−1^, Table 1. Carotenoid levels and profiles in non-green tissues e.g., mushroom tissues can vary greatly and are regulated by a variety of factors, including developmental stage, environment, stress, or a combination of these [47]. As metabolites with a protective effect, they are among the most efficient physical quenchers of singlet oxygen (^1^O_2_), a highly reactive species, due to their energy levels being near to that of ^1^O_2_ [36,66]. Furthermore, as a potential ^1^O_2_ quencher, lycopene was demonstrated to pass the blood-brain barrier and be present in the central nervous system in low amounts [67]. As food additives, lycopene and β-carotene may also be used as a chain-breaking antioxidant in a lipid environment, especially under low oxygen partial pressure. The extensive systems of double bonds make them susceptible to attack •LOO, resulting in the formation of inactive products [36]. Among investigated polypore extracts, *F. pinicola* and *G. lucidum* can be considered excellent sources for β-carotene supplementation, i.e., vitamin A. According to data from the Mayo Clinic, if β-carotene is taken as a supplement the adequate daily dose for teenagers and adults is between 6 and 15 mg (the equivalent of 10,000 to 25,000 Units of vitamin A activity) [68,69]. Moreover, a strong correlation between β-carotene content and inhibition of LPx of investigated extracts confirmed their potential as a chain-breaking antioxidant in a lipid environment. Additionally, very strong correlation was observed between lycopene content and the chelating ability of Fe^2+^ as well as a strong correlation between β-carotene content and the chelating ability of Fe^2+^ (Figure 3 and Table S1). Because Fe^2+^ are the most powerful pro-oxidants in the food system [70], the investigated polypore mushroom extracts might be applied to improve food quality.

Furthermore, a strong correlation was observed between the total content of β-carotene and tyrosinase inhibition (Figure 4, Table S2). Anantharaman et al. indicated that apocarotenoids may inhibit tyrosinase activity in a dose-dependent manner [71]. Moreover, their molecular docking results implied that apocarotenoids were allosterically bound to tyrosinase through hydrophobic interactions [71].

There is not much research related to the anti-tumor activities of carotenoids. Preclinical data suggest that carotenoids might reduce growth or induce apoptosis in cancer cells [72,73]. An example is the research of Gloria et al. on the effect of carotenoids on the cell cycle and cell viability in human breast adenocarcinoma cancer cell lines (MCF-7, MDA-MB-231, and MDA-MB-235) [72]. This research indicated that the effect of lycopene and β-carotene on cancer cells was cell type-dependent. In contrast to these findings, our study confirmed that β-carotene and lycopene were in the highest concentrations in *F. pinicola* extract which possessed the highest cytotoxicity against breast carcinoma MDA-MB-453 cells, as well as against K562 and HeLa cells, Table 4. It should also be taken into account that these molecules were present in a significantly lower concentration compared to the TPC in *F. pinicola* extract (Table 1). Accordingly, potential synergistic and/or agonistic effect among these molecules should be investigated in expression of the anti-tumor activities [74].

### 3.3. Vitamin C

Vitamin C was found in all of the extracts examined (Table 1). The influence of location and environmental conditions of growth on the content of vitamin C in mushrooms has been insufficiently investigated so far. It can be speculated that the factors affecting the content of vitamin C in mushrooms may be the same as for plants such as genotypic differences, and climatic conditions e.g., the higher the intensity of light during the growing season, the greater vitamin C content in tissues; maturity and harvesting methods, and postharvest handling procedures [75]. It is an essential nutrient for humans [76]. Likewise, vitamin C is a normal skin constituent found at high levels in both the dermis and epidermis [76]. Vitamin C has been shown as an effective RS scavenger. It may also act as a secondary antioxidant, e.g., recycling vitamin E, the main lipid-soluble antioxidant [24,36]. In contrast to these findings, regression analysis in our study did not reveal a significant correlation between vitamin C content and SA_•DPPH_, SA_•OH_, FRAP, and LPx assays (Figure 3, Table S1). A strong and negative correlation was observed with EC_50_ values in the chelating ability of Fe^2+^ (Figure 3). It was in opposition to the other constituents of the extracts, polyphenols, and carotenoids, which primarily react as radical scavengers and as effective inhibitors of LPx (Figure 3, Table S1). These results were consistent with the results obtained from correlation analysis among the antioxidant assays (Table S1). Accordingly, it could be concluded that polypore methanol extracts may act as antioxidants in mixed mode. As primary antioxidants (chain breaking, RS scavengers) they can directly scavenge RS by direct reduction via electron transfers or by radical quenching via H atom transfer, or by acting in the mixed mechanism. As secondary antioxidants, they may be involved in the deactivation of metals (Fe^2+^ chelating ability), and the inhibition or breakdown of lipid hydroperoxides. The significance of vitamin C in the expression of the chelating ability of Fe^2+^ may be important in the explanation of some cytotoxicity mechanisms against malignant cell lines in this study. Tumor cells contain more iron than other normal tissues [77,78]. Thus, it is reasonable to expect that vitamin C may influence apoptosis in vitro in this study [77,78,79].

From regression analysis, it can also be concluded that vitamin C content in this study does not have significant influence on the inhibition of tyrosinase and ACE, Figure 4, Table S2.

## 4. Materials and Methods

### 4.1. Collection, Identification, and Drying of Mushroom Fruiting Bodies

**Figure 6:**
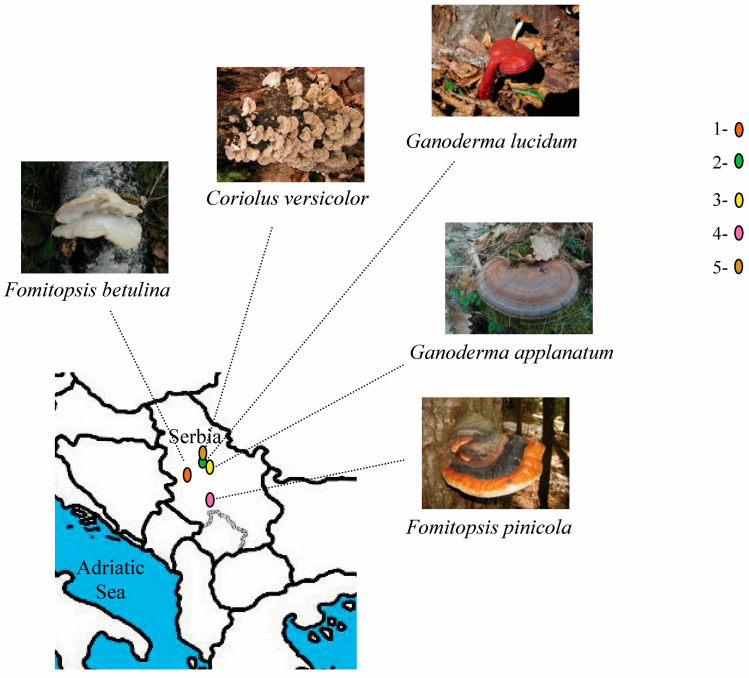
Mushrooms sampling sites; (1) Divčibare, (2) Avala, (3) Babe, (4) Kopaonik, (5) Košutnjak.

Fresh wild-growing fruiting bodies of the five Polypore mushroom species, namely *F. betulina*, *F. pinicola*, *G. applanatum*, *G. lucidum*, and *C. versicolor* were collected from several sites/locations in the different regions of the Republic of Serbia (Figure 6, detailed data in Supplementary Materials—Table S4). Carpophores of each mushroom species were identified according to the methods of classical herbarium taxonomy to confirm the correct species [80,81]. Representative voucher specimens were deposited in the herbarium of the Department for Industrial Microbiology at the Faculty of Agriculture, University of Belgrade, together with their mycelia cultures. For further analysis, collected mushrooms were cut into thin slices and lyophilized (Christ BETA 2–8 LD plus freeze dryer, Osterode, Germany), powdered, and subjected to methanol extraction. The solvent of choice (methanol) favors the extraction of compounds analyzed in this study [82].

### 4.2. Preparation of Extracts

The dry sample (5 g) was extracted by stirring with 100 mL of methanol at 120 rpm for 24 h and filtered through Whatman No. 4 paper. The residue was then extracted with two additional 100 mL portions of methanol, as described earlier [83]. The combined methanol extracts were evaporated at 40 °C to dryness (Buchi Rotavapor^®^R II) and stored at 4 °C for further use.

### 4.3. Secondary Metabolites Analysis

#### 4.3.1. Total Polyphenol Content (TPC)

The Folin—Ciocalteu reaction method adapted for a 96-well microplate reader (Hanna Instruments EC 215 Conductivity Meter, Chelmsford, UK) was used to determine the TPC in methanol extracts of mushrooms [84]. Gallic acid was used as standard (0.015–0.25 mg mL^−1^) and results were expressed as mg of gallic acid equivalent (GAE) per g of extract dry weight (DW).

#### 4.3.2. Vitamin C Content

The method adjusted for a 96-well microplate assay was previously described [82]. All samples were analyzed in triplicates and calculated based on the standard curve of vitamin C (concentration range: 0.03125–2 mg mL^−1^). The results are expressed as mg g^−1^ of ascorbic acid (based on extract DW).

#### 4.3.3. Determination of β-Carotene and Lycopene Content

β-Carotene and lycopene content were determined according to the method of Nagata & Yamashita [83,85]. The dried methanol extract (100 mg) was vigorously shaken with 10 mL of an acetone–hexane mixture (4:6) for 1 min and filtered through Whatman No. 4 filter paper. The absorbance of the filtrate was measured at 453, 505, and 663 nm. Contents of β-carotene and lycopene were calculated according to the following equations: lycopene (mg/100 mL) = −0.0458A_663_ + 0.372A_505_ − 0.0806A_453_; β-carotene (mg/100 mL) = 0.216A_663_ − 0.304A_505_ + 0.452A_453_. The results are expressed as μg of carotenoid per g of the extract DW.

#### 4.3.4. LC-MS/MS of Phenolics

Samples and standards were analyzed using a 1260 Series liquid chromatography system (Agilent Technologies, Santa Clara, CA, USA) coupled with a 6460A Triple Quad tandem mass spectrometer (Agilent Technologies, Santa Clara, CA, USA) with an electrospray ion source (Agilent Technologies) by the analytical conditions reported in an earlier study [86]. This coupled system was controlled using MassHunter software (version B.06.00, Agilent Technologies). Authentic polyphenol standards were: gallic acid, protocatechuic acid, p-hydroxybenzoic acid, chlorogenic acid, syringic acid, vanillic acid, caffeic acid, p-coumaric acid, ellagic acid, trans-cinnamic acid, ferulic acid, vanillin, quercetin, rutin, catechin, kaempferol, and resveratrol.

### 4.4. Determination of Antioxidant Potential

Five methods were used to evaluate radical-blocking capacities: 1,1-diphenyl-2-picrylhydrazyl free radical (•DPPH) scavenging ability (SA_•DPPH_) [40,83], inhibition of lipid peroxidation (LPx) in a linoleic acid model system [40,83], ferric-reducing antioxidant power (FRAP) assay [40,83], ferrous-ion chelating ability [40,83], and hydroxyl radical (•OH) scavenging ability (SA•OH) [36]. •OH was generated in the Fenton reaction system by mixing 200 μL of 112 mM DMPO (5,5-dimethyl-1-pyrroline-N-oxide), 200 μL of DMF (N, N- dimethylformamide), 200 μL of 2 mM H_2_O_2_, and 2 μL of 30 mM Fe^2+^ (control). The stabilization of •OH was investigated by the ESR spin trapping method [36,87]. The ESR spectra were recorded after 2.5 min, with the following spectrometer settings: field modulation 100 kHz, modulation amplitude 0.226 G, receiver gain 5 × 105, time constant 80.72 ms, conversion time 327.68 ms, center field 3440.00 G, sweep width 100.00 G, x-band frequency 9.64 GHz, power 20 mW and temperature 23 °C. SA_•OH_ of mushroom extracts dissolved in DMF was defined as SA•OH (%) = 100 × (h0 − hx)/h0, where h0 and hx are the heights of the second peak in the ESR spectrum of 5,5-dimethyl-1-pyrroline-N-oxide (DMPO)-OH spin adduct of the control and the sample, respectively.

In all assays, the results were expressed as: % of •DPPH and % of •OH scavenging activity, % of inhibition of linoleic acid peroxidation, % of Fe^2+^ chelation, and increase in absorbance at 700 nm in the measurement of reduction ability. Additionally, results were expressed as EC_50_ values, the concentration (or dose) effective in producing 50% of the maximal response, which is a convenient way of comparing the potencies of drug or supplements. In this study, EC_50_ values represent the effective concentrations of each extract (mg mL^−1^) required to show 50% antioxidant activity and was obtained by interpolation from linear regression analysis. In order to more adequately compare the chelating ability of the examined extracts, the results were also expressed as mM of Fe^2+^ equivalents per gram of extract dry weight (mM Fe^2+^ g^−1^ extract dw). The results of EC_50_ values in chelating ability (mg mL^−1^) obtained from the appropriate reaction conditions and an initial ferric chloride concentration of 2 mM [40] were converted into Fe^2+^ equivalents.

Extracts were analyzed at the concentration range of 0.01–0.5 mg mL^−1^ for •OH scavenging ability, 0.001–0.1 mg mL^−1^ for inhibition of LPx in the linoleic acid model system, and 0.01–5 mg mL^−1^ for all other antioxidant assays. Ascorbic acid, α-tocopherol acetate, citric acid, and ethylenediaminetetraacetic acid (EDTA) were used as positive controls. They are common additives in food formulations and are typically used at mg levels.

### 4.5. Enzyme Inhibition Activities

Tyrosinase inhibitory potential of the tested extracts was determined in the reaction solution of 46 units/mL tyrosinase (EC 1.14.18.1, Merck) and 2.5 mM of L-DOPA in a 96-well plate using an absorbance microplate reader (ELx808, BioTek Instruments, Inc., Winooski, VT, USA) controlled by Gen5^TM^ Software [57]. Extracts were analyzed at the concentration range of 0.031–4.0 mg mL^−1^, using kojic acid as a reference. The inhibition rate of tyrosinase was calculated according to the following formula:

Inhibition (%) = [1 − (C − D)/(A − B)] *×* 100 (1)

where A is the absorbance of each well without testing extracts, B is blank (the absorbance without testing extract and L-DOPA), C is the absorbance of wells with testing extracts, and D is the absorbance with testing extracts but without tyrosinase.

Angiotensin-converting enzyme (ACE) activity was analyzed using the method described in a previous investigation [9]. Briefly, the inhibition percentage of extracts was determined by replacing 30 μL of water with the same volume of the sample to be studied in the concentration range 0.005–5.0 mg mL^−1^ in the reaction solution of 26 mU mL^−1^ ACE (EC 3.4.15.1, Merck). After the addition of 10 mM hippuryl-His-Leu (HHL) (200 μL) dissolved in 0.2 M phosphate buffer (pH 8.3), the reaction solution was incubated at 37 °C for 80 min. ACE activity was stopped by a decrease in pH by the addition of 1 M HCl (150 μL). The hippuric acid formed in the enzymatic process was extracted with 1 mL of ethyl acetate, shaken, and then centrifuged at 3000× *g* for 10 min. The aliquot of the organic layer was taken and evaporated under airflow at 37 °C. The residue, hippuric acid, was redissolved in 1 mL of MQ water, and the absorbance was measured at 228 nm. A solution of captopril (1 mg mL^−1^) was used as the positive control. One tablet containing 25 mg of captopril was pulverized in a mortar and extracted with 25 mL of distilled water in an ultrasonic bath. Water extract was filtered through a filter with a pore size of 0.45 μm. Extracts contained substances that could interfere, and it was necessary to determine their absorbance and apply the formula provided in this study [9]. The sample blank was prepared in the same way as the reaction blank, replacing the volume of water with the sample. The inhibition rate of ACE was calculated using equation 1, as for the calculation of anti-tyrosinase activity, where A represents absorbance in the presence of ACE, B is the absorbance of the reaction blank, C is the absorbance in the presence of ACE and inhibitor (captopril or extract), and D is the absorbance of the sample blank.

The enzyme inhibitory activities in both assays were expressed as inhibition percentage and IC_50_ values, which were calculated using linear regression analyses, as the concentration of extract required for 50% in vitro inhibition.

### 4.6. In Vitro Cytotoxic Activity

Stock solutions of methanol mushroom extracts were prepared in dimethyl sulfoxide (DMSO) and afterward diluted with a complete nutrient medium (RPMI-1640) as described in our previous report [83]. Human cervix adenocarcinoma (HeLa), breast carcinoma (MDA-MB-453), normal lung fibroblast (MRC-5), and normal human bronchial epithelial (BEAS-2B) cells were cultured as monolayers in the nutrient medium, while myelogenous leukemia (K562) cells were maintained as a suspension culture. The cells were grown at 37 °C in a 5% CO_2_ and humidified air atmosphere. Cell lines were obtained from the American Type Culture Collection (ATCC, Manassas, VA, USA). For the cell sensitivity analysis, HeLa (2.000 cells per well), MDA-MB-453 (3.000 *c*/*w*), MRC-5, and BEAS-2B (5.000 *c*/*w*) cells were seeded into 96-well microtiter plates and 20 h later different concentrations (0.0156–2 mg mL^−1^) of investigated extracts were appended to the wells. Investigated compounds were added to a suspension of K562 cells (5.000 *c*/*w*) 2 h after cell seeding, in the same final concentrations applied to other cells. Cell survival was determined by the 3-(4,5-dimethylthiazol-2-yl)-2,5-diphenyl tetrazolium bromide (MTT) test according to the method of Ohno and Abe (1991), 72 h after the investigated extracts were added. Concentrations of the extracts that induced a 50% decrease in malignant and normal cell survival (IC_50_ values) were calculated from a dose-response growth curve. Doxorubicin was used as a control.

#### Selectivity Coefficient in the Cytotoxic/Anti-Tumor Action

To evaluate further the anticancer potential of the extracts, selectivity in cytotoxic/anti-tumor action against specific malignant cell lines, in comparison to normal MRC-5 and BEAS-2B cells, was also determined. The selectivity coefficient (*SC*) was calculated by the following equation: SC = IC_50_ normal cells/IC_50_ cancer cells.

### 4.7. Statistical Analysis

All experiments were carried out in triplicate and expressed as the mean ± standard deviation (SD). Statistical analyses were performed with the Statistica 12.0 software package (StatSoft Inc., Tulsa, OK, USA), using a one-way analysis of variance (ANOVA) for all collected data. Differences between the means for each treatment were determined using Duncan’s multiple range tests (*p* < 0.05). The correlation coefficients (*r*) between antioxidant activities and methanol extracts as well as enzyme inhibition activities and methanol extracts were determined.

## 5. Conclusions

Due to the high levels of phenolic compounds, vitamin C, and carotenoids, polypore mushroom methanol extracts of the five lignicolous species *F. betulina*, *F. pinicola*, *G. applanatum*, *G. lucidum*, and *C. versicolor* from various regions of Serbia, the Western Balkan region, have high biological/industrial potential and should be considered for spawn development. Furthermore, in the functional food market, the commercialization of wild mushroom species with potential biological activity is a growing trend. The extracts tested positive for suppression of LPx in the linoleic acid model system as well as inhibition of the detrimental propagation of radical chain reactions. All extracts had strong anti-tyrosinase activity and modest ACE inhibition. The most promising actions were identified for *F. pinicola* extract, which corresponded to the highest values of the total amount of secondary metabolites. Furthermore, *F. pinicola* had the best activity against the selected malignant cell lines that met the NCI cytotoxicity criteria for crude extracts. Otherwise, *G. lucidum* extract displayed considerably stronger selectivity in anti-tumor action against malignant cell lines, mainly MRC-5, which are involved in connective tissue production, when compared to normal cells. The content of bioactive compounds like phenolics is frequently determined by the location and environmental conditions under which they grow. Thus, screening and adopting local strains can offer higher quality material to use in the food, pharmaceutical, and agricultural sectors. At the same time, this practice supports the preservation of the local pool of fungi species.

As observed and emphasized in this study, the environmental conditions, substrate, climate, stage of development, and species affect bioactive molecules content, diversity, and biological activity. Future studies are directed to consider these factors and determine the exact species-selected factors relations.

One should keep in mind that there are considerable changes in the activities of the metabolic form of bioactive molecules and their form in the mushroom. Thus, potential candidates should be isolated and tested in real systems like food or dietary supplements and further strengthened by clinical studies. In this way, promising activities like the inhibition of tyrosinase, antihypertensive, or antioxidant activity may or may not be confirmed which will strengthen the market position of this material and build trust among consumers.

## Figures and Tables

**Table 1:** Extraction yields and secondary metabolite contents in methanol extracts of the five lignicolous mushroom species.

Extract	Yield (mg g^−1^) ^α^	Total Content (mg g^−1^) ^β^		Total Content (μg g^−1^) ^β^	
		Phenols ^δ^	Vitamin C	β-Carotene	Lycopene
*F. betulina*	260.5 ± 15.3 ^B^*^γ^*	32.6 ± 2.1 ^C^	1.9 ± 0.1 ^B^	51.7 ± 6.2 ^E^	23.8 ± 2.6 ^E^
*F. pinicola*	360.3 ± 20.1 ^A^	133.1 ± 6.2 ^A^	2.0 ± 0.2 ^B^	541.5 ± 21.1 ^A^	265.7 ± 13.1 ^A^
*G. applanatum*	120.2 ± 10.3 ^C^	39.4 ± 1.8 ^B^	1.2 ± 0.1 ^C^	188.6 ± 13.5 ^C^	57.5 ± 4.7 ^D^
*G. lucidum*	100.8 ± 7.9 ^D^	38.6 ± 2.6 ^B^	3.0 ± 0.1 ^A^	372.2 ± 18.9 ^B^	224.6 ± 17.3 ^B^
*C. versicolor*	60.7 ± 5.8 ^E^	10.5 ± 0.9 ^D^	3.1 ± 0.2 ^A^	158.7 ± 10.0 ^D^	109.2 ± 8.2 ^C^

^α^ All values are on a dry weight (DW) basis of the fruiting body; ^β^ All values of metabolites are on a DW basis of extract; data represent mean ± standard deviation (*n* ± 3); *^γ^* within the same column, means followed by different letters are significantly different at *p* ≤ 0.05; ^δ^ gallic acid equivalents (GAE).

**Table 2:** Identification and quantification of phenolic compounds in methanol extracts of the five lignicolous mushroom species by LC-MS/MS.

Compounds	*F. betulina*	*F.pinicola*	*G. applanatum*	*G.lucidum*	*C.versicolor*
Simple phenols/ derivative (μg g^−1^)					
Vanillin	n.d.	1.06 ± 0.02 ^B*γ*^	1.81 ± 0.07 ^A^	0.81 ± 0.01 ^C^	0.83 ± 0.02 ^C^
Hydroxybenzoic acids (μg g^−1^)					
Gallic acid	1.48 ± 0.05 ^C^	951.12 ± 6.30 ^A^	1.93 ± 0.06 ^B^	0.94 ± 0.01 ^D^	0.77 ± 0.01 ^E^
Ellagic acid	n.d.	31.23 ± 1.22 ^A^	22.03 ± 1.01 ^B^	n.d.	n.d.
Protocatechuic acid	29.94 ± 0.09 ^A^	11.62 ± 0.62 ^B^	8.92 ± 0.31 ^C^	5.26 ± 0.11 ^E^	6.72 ± 0.13 ^D^
p-Hydroxybenzoic acid	94.27 ± 1.11 ^A^	7.59 ± 0.13 ^D^	12.76 ± 0.08 ^B^	11.42 ± 0.07 ^C^	0.24 ± 0.01 ^E^
Vanillic acid	11.20 ± 0.07 ^B^	n.d.	19.80 ± 0.10 ^A^	n.d.	n.d.
Syringic acid	6.37 ± 0.22 ^B^	3.07 ± 0.09 C	9.34 ± 0.12 ^A^	n.d.	n.d.
Hydroxycinnamic acids (μg g^−1^)					
Trans-cinnamic acid	n.d.	n.d.	n.d.	n.d.	n.d.
Chlorogenic acid	1.33 ± 0.02 ^A^	0.76 ± 0.01 ^B^	0.75 ± 0.02 ^B^	0.36 ± 0.01 ^C^	n.d.
Caffeic acid	n.d.	n.d.	n.d.	3.73 ± 0.10	n.d.
p-coumaric acid	0.49 ± 0.01 ^C^	n.d.	0.79 ± 0.01 ^B^	18.24 ± 0.92 ^A^	n.d.
Ferulic acid	LOD	LOD	LOD	LOD	LOD
Sinapic acid	LOD	LOD	LOD	LOD	LOD

nd: not detected; LOD: limit of detection—the smallest amount or concentration of the analyte in the test sample that can be reliably distinguished from zero; The LOD value was set at the level of 0.005 µg mL^−1^ for all phenolic compounds. All values of metabolites are on a dry weight (DW) basis of extract; data represent mean ± standard deviation (*n* ± 3); *^γ^* within the same row, means followed by different letters are significantly different at *p* ≤ 0.05.

**Table 3:** EC_50_ and IC_50_ values of polypore mushroom methanol extracts in the antioxidant potential and enzyme inhibition assays.

Property	*F. betulina*	*F. pinicola*	*G. applanatum*	*G. lucidum*	*C. versicolor*
Antioxidant potential EC_50_ (mg mL^−1^)					
Inhibition of LPx	0.091 ± 0.003 ^C *γ*^	0.012 ± 0.001 ^A^	0.018 ± 0.001 ^A^	0.036 ± 0.004 ^B^	0.096 ± 0.002 ^C^
SA_•DPPH_	1.51 ± 0.11 ^E^	0.49 ± 0.05 ^A^	0.048 ± 0.006 ^B^	0.88 ± 0.08 ^C^	0.68 ± 0.09 ^D^
SA_•OH_	0.23 ± 0.01 ^B^	0.05 ± 0.005 ^A^	0.06 ± 0.005 ^A^	0.29 ± 0.02 ^C^	0.36 ± 0.03 ^C^
FRAP	3.51 ± 0.16 ^E^	2.37 ± 0.13 ^D^	0.30 ± 0.02 ^A^	1.36 ± 0.09 ^B^	1.92 ± 0.12 ^C^
Fe^2+^ chelating ability	4.78 ± 0.31 ^D^	0.99 ± 0.09 ^A^	4.13 ± 0.10 ^C^	1.17 ± 0.07 ^B^	1.02 ± 0.09 ^A,B^
Enzyme inhibition IC_50_ (mg mL^−1^)					
Tyrosinase inhibitory activity	0.69 ± 0.05 ^C^	0.10 ± 0.04 ^A^	0.51 ± 0.08 ^B^	0.81 ± 0.07 ^C^	1.16 ± 0.09 ^E^
ACE inhibitory activity	1.01 ± 0.12 ^B^	0.65 ± 0.04 ^A^	0.89 ± 0.09 ^B^	1.28 ± 0.11 ^C^	1.96 ± 0.18 ^D^

Data represent mean ± standard deviation (*n* ± 3); *^γ^* within the same row, means followed by different letters are significantly different at *p* ≤ 0.05; EC_50_ value represents the effective concentration of extract required to show 50% antioxidant activities; IC_50_ value represents the effective concentration of extract required for 50% in vitro enzyme inhibition.

**Table 4:** IC_50_ values of lignicolous mushroom methanol extracts in the cytotoxicity against human malignant cells and non-cancer human cells.

Cytotoxicity IC_50_ (µg mL^−1^)	*F. betulina*	*F. pinicola*	*G. applanatum*	*G.lucidum*	*C. versicolor*
Human malignant cells					
HeLa	42 ± 4 ^B *γ*^	24 ± 5 ^A^	60 ± 12 ^C^	164 ± 20 ^D^	232 ± 23 ^E^
K562	40 ± 2 ^B^	29 ± 3 ^A^	76 ± 10 ^C^	148 ± 64 ^D^	220 ± 29 ^D^
MDA-MB-453	42 ± 1 ^B^	23 ± 2 ^A^	66 ± 13 ^C^	170 ± 2 ^D^	251 ± 21 ^E^
Non-cancer human cells					
MRC-5	43 ± 5 ^A^	32 ± 6 ^A^	83 ± 6 ^B^	360 ± 10 ^C^	402 ± 21 ^D^
BEAS-2B	40 ± 7 ^A^	29 ± 4 ^A^	79 ± 6 ^B^	230 ± 20 ^C^	280 ±13 ^D^

Data represent mean ± standard deviation (*n* ± 3); ^γ^ within the same row, means followed by different letters are significantly different at *p* ≤ 0.05; IC_50_ value representing the effective concentration of extract required for 50% in vitro inhibition.

## Data Availability

The data presented in this study are available on request from the corresponding author.

## Supplementary Materials

The following supporting information can be downloaded at: https://www.mdpi.com/article/10.3390/molecules29020314/s1, Table S1: Relationship between EC_50_ values in antioxidant activities and analyzed secondary metabolite content; Table S2: Relationship between IC_50_ values in enzyme inhibition and analyzed secondary metabolite content; Table S3: Selectivity of methanol extracts in anti-tumor action; Table S4: Mushroom species collected with corresponding family, habitat, sampling locations and usability, according to the map in Figure 1. Figure S1: Total chromatograms (TIC) of all samples and one calibration sample (*F. pinicola*-uz 1, *F.betulina*-uz 2, *G. applanatum*-uz 3, *G. lucidum*-uz 4, *C. versicolor* –uz 5). Figure S2: Multiple reaction monitoring (MRM) transitions of polyphenols in analyzed samples (*F. pinicola* –uz 1, *F. betulina*-uz 2, *G. applanatum*-uz 3, *G. lucidum*-uz 4, *C. versicolor* –uz 5).
