# Supplementary material for: Bioprospecting of Selected Species of Polypore Fungi from the Western Balkans
Source: Molecules. 2024 Jan 8;29(2):314. doi: 10.3390/molecules29020314 (PMC10819588; doi:10.3390/molecules29020314)
Supplement: Supplementary file 1 [file molecules-29-00314-s001.zip › molecules-2764930-supplementary.pdf]

Supplementary information

Bioprospecting of Selected Species of Polypore Fungi from the Western Balkans

Maja Kozarski [1], Anita Klaus [1], Bojana Špirović-Trifunović [2], Srdjan Miletić [3], Vesna Lazić [1], Željko Žižak [4] and Jovana Vunduk [5,*]

[1] Institute for Food Technology and Biochemistry, Faculty of Agriculture, University of Belgrade, Nemanjina 6, 11080 Belgrade, Serbia; maja@agrif.bg.ac.rs (M.K.); aklaus@agrif.bg.ac.rs (A.K.); vlazic93@gmail.com (V.L.)
[2] Faculty of Agriculture, University of Belgrade, Nemanjina 6, 11080 Belgrade, Serbia; spirovic@agrif.bg.ac.rs (B.Š.-T.)
[3] Institute of Chemistry, Technology and Metallurgy, University of Belgrade, Njegoševa 12, 11000 Belgrade, Serbia; srdjan.miletic@ihtm.bg.ac.rs (S.M.)
[4] Institute of Oncology and Radiology of Serbia, Paterova 14, 11000 Belgrade, Serbia; zizakz@ncrc.ac.rs (Ž.Ž.)
[5] Institute of General and Physical Chemistry, Studentski trg 12/V, 11158 Belgrade, Serbia; jvunduk@iofh.bg.ac.rs (J.V.)
* Correspondence: jvunduk@iofh.bg.ac.rs; Tel.: +381 64 020 9819

Table S1. Relationship between EC_{50} values in antioxidant activities and analyzed secondary metabolite content.

	Inhibition of LPx	SA•DPPH	SA•OH	FRAP	Fe^{2+} chelating ability
Inhibition of LPx	1	0.68	0.76	0.57	0.17
SA•DPPH	0.68	1	0.62	0.85	0.23
SA•OH	0.76	0.62	1	0.26	0.26
FRAP	0.57	0.85	0.26	1	0.13
Fe^{2+} chelating ability	0.17	0.23	-0.21	0.13	1
TPC	-0.68	-0.25	-0.70	0.16	-0.30
Vitamin C	0.45	0.35	0.83	0.17	-0.71
β-carotene	-0.74	-0.37	-0.40	-0.16	-0.69
likopene	-0.56	-0.21	-0.11	-0.078	-0.85

Correlation coefficient (*r*)– all values are statistically significant ($P<0.05$); for absolute values of *r*, 0-0.19 is regarded as a 'very weak', 0.2-0.39 as a 'weak', 0.40-0.59 as a 'moderate', 0.6-0.79 as a 'strong', and 0.8-1 as a 'very strong' correlation.

Table S2. Relationship between IC_{50} values in enzyme inhibition and analyzed secondary metabolite content

	Tyrosinase inhibitory activity	ACE inhibitory activity
TPC	-0.88	-0.71
Vitamin C	0.66	0.78
β-carotene	-0.60	-0.39
likopen	-0.38	-0.15

*r** – all values are statistically significant ($p \leq 0.05$); for absolute values of *r*, 0-0.19 is regarded as a 'very weak', 0.2-0.39 as a 'weak', 0.40-0.59 as a 'moderate', 0.6-0.79 as a 'strong', and 0.8-1 as a 'very strong' correlation

Table S3. Selectivity of methanol extracts in antitumor action.

SC[α] in the antitumour action [IC_{50} (normal-human cells)/ IC_{50} (human malignant cells)]	*F.betulina*	*F. pinicola*	*G. applanatum*	*G. lucidum*	*C. versicolor*
MRC-5/HeLa	1.02	1.33	1.38	2.20	1.73
MRC-5/ K562	1.08	1.10	1.10	2.43	1.83
MRC-5/MDA-MB-453	1.02	1.39	1.26	2.12	1.60
BEAS-2B/HeLa	0.95	1.21	1.32	1.83	1.21
BEAS-2B/ K562	1.00	1.00	1.04	1.55	1.28
BEAS-2B/ MDA-MB-453	0.95	1.26	1.20	1.35	1.12

[α]Selectivity coefficient

Table S4. Mushroom species collected with corresponding family, habitat, sampling locations and usability. According to the map of Figure 1.

Species	Family	Habitat	Sampling location	Usability/edibility
Fomitopsis betulina	Fomitopsidaceae	Brown rot fungi, common bracket, mainly on birch trees	1-Divcibare, resort on the mountain Maljen (1,104 m), western Serbia; 44° 5'51.68"N 19°59'39.77"E	Medicinal/good
Ganoderma lucidum	Ganodermataceae	White rot fungi, on decaying hardwood trees	2-Avala, mountain (511 m), near Belgrade, Serbia; 44°41'29.93"N 20°30'34.09"E	Medicinal/hard
Ganoderma applanatum	Ganodermataceae	White rot fungi, causes a rot of heartwood of deciduous and coniferous trees	3-village Babe, mountain Kosmaj (626 m), south of Belgrade, Serbia 44°32'4.36"N 20°30'10.41"E	Medicinal/hard
Fomitopsis pinicola	Fomitopsidaceae	Brown rot fungi, causes a stem decay on softwood and hardwood trees	4-Kopaonik, mountain (2,017m), national park, south Serbia 43°18'13.45"N 20°45'55.52"E	Medicinal/hard
Coriolus versicolor	Polyporaceae	White rot fungi, in groups on logs and stumps of deciduous trees	5- Kosutnjak, a large forest area, Belgrade, Serbia 44°45'37.48"N 20°26'24.86"E	Medicinal/good

Figure S1. Total chromatograms (TIC) of all samples and one calibration sample (*F. pinicola*-uz 1, *F.betulina*-uz 2, *G. applanatum*-uz 3, *G. lucidum*-uz 4, *C. versicolor* –uz 5)

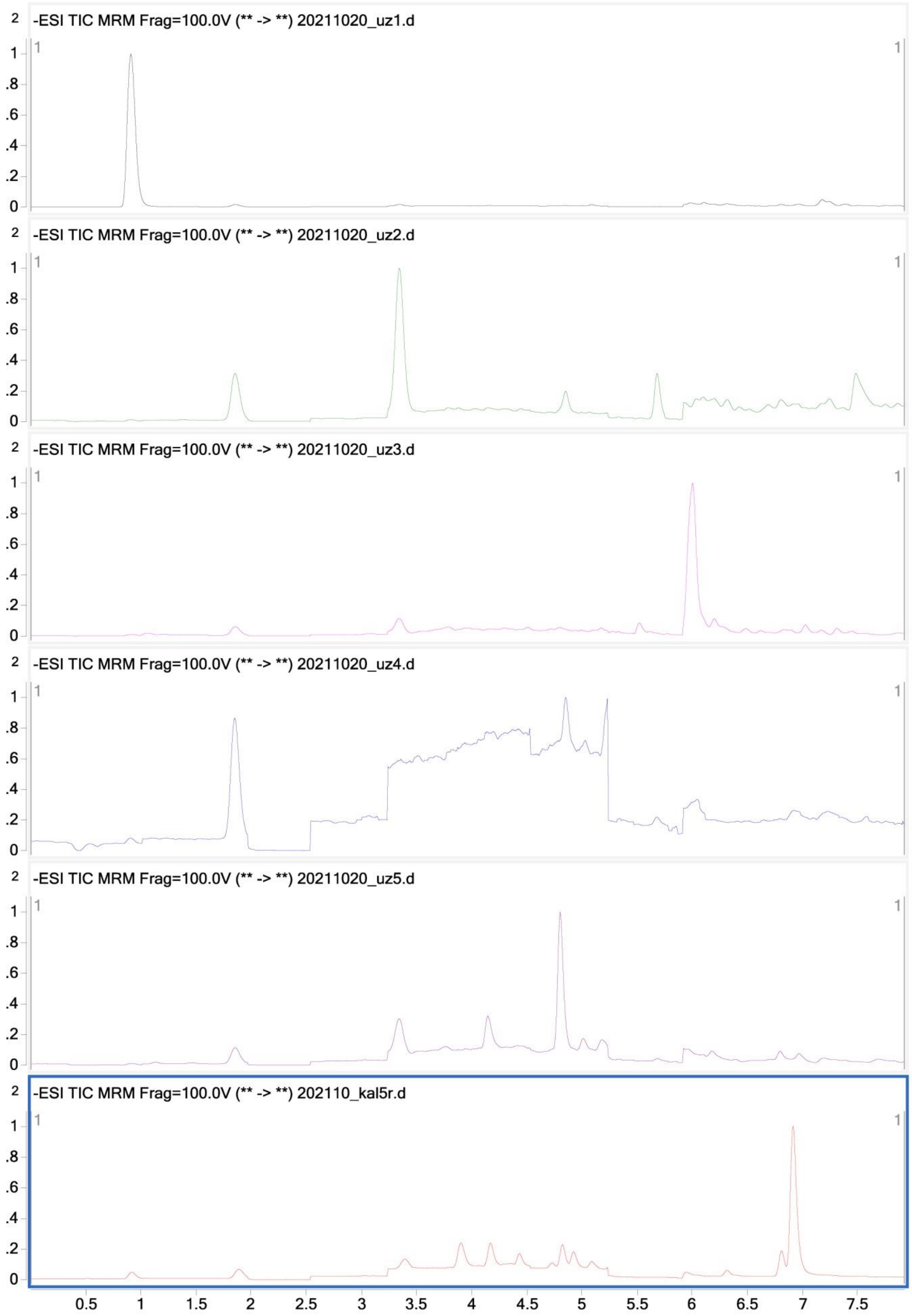

Figure S2. Multiple reaction monitoring (MRM) transitions of polyphenols in analyzed samples

(*F. pinicola* –uz 1, *F. betulina*-uz 2, *G. applanatum*-uz 3, *G. lucidum*-uz 4, *C. versicolor* –uz 5)

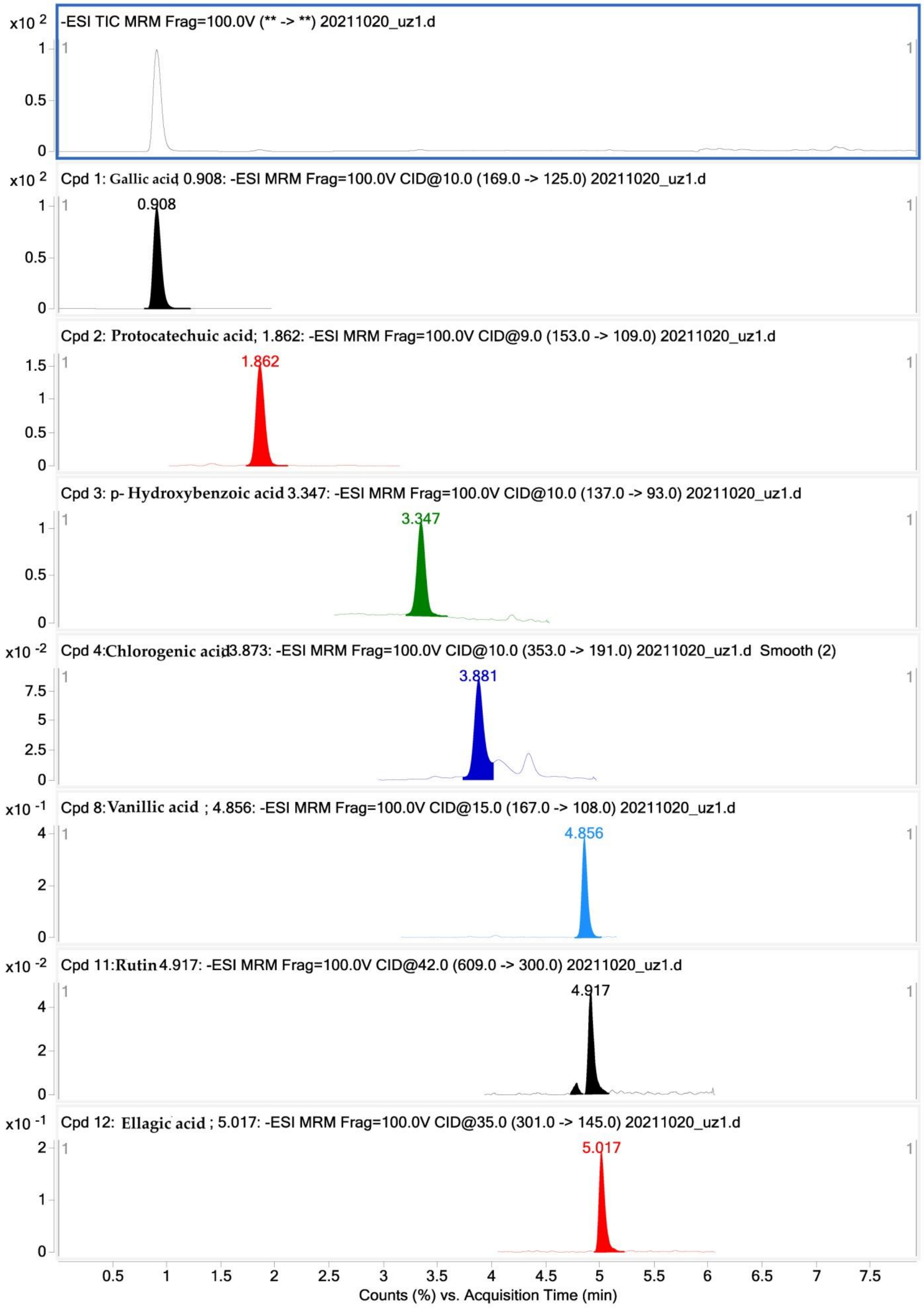

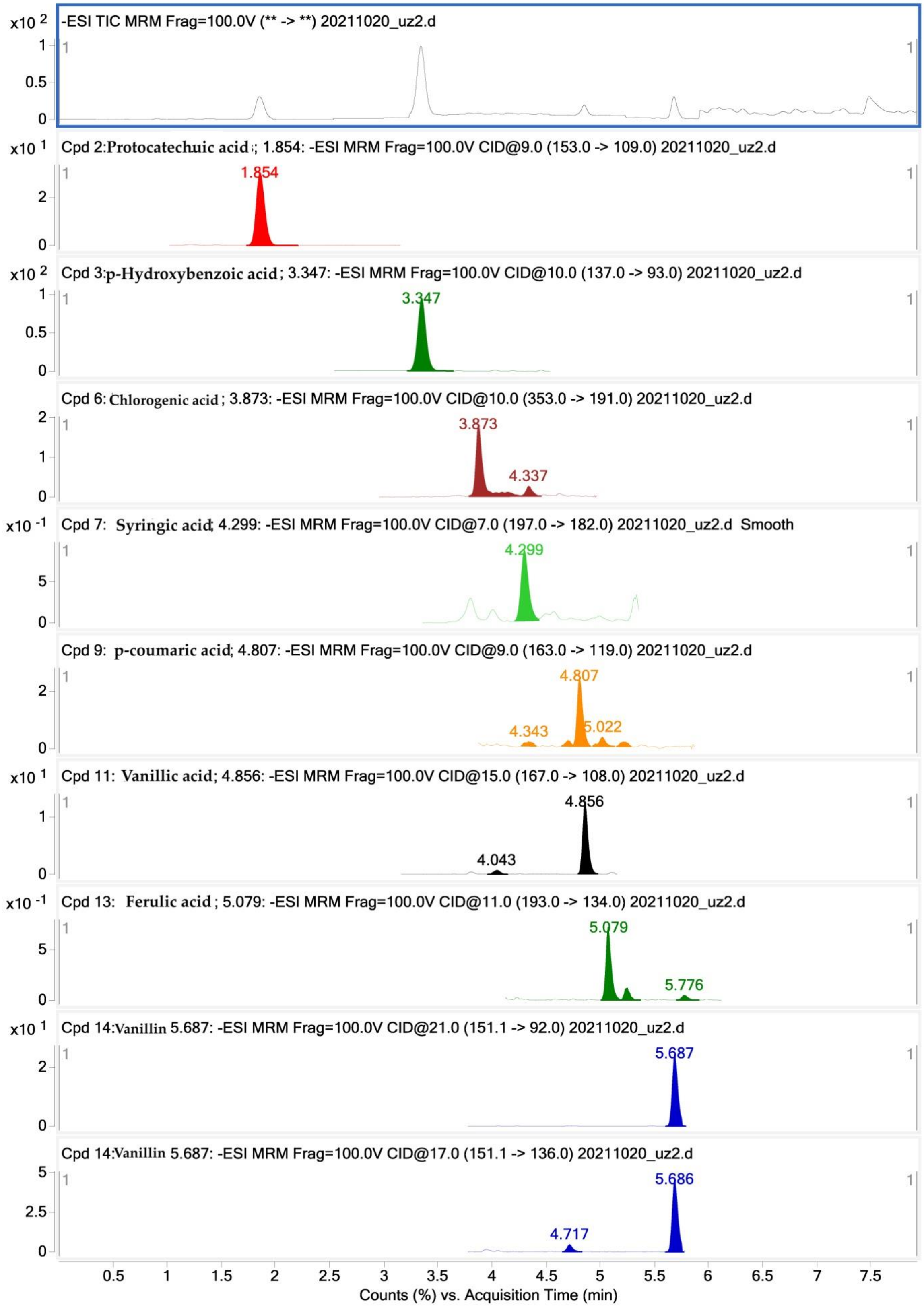
-ESI TIC MRM Frag=100.0V (** -> **) 20211020_uz2.d
Cpd 2:Protocatechuic acid ; 1.854: -ESI MRM Frag=100.0V CID@9.0 (153.0 -> 109.0) 20211020_uz2.d
1.854
Cpd 3:p-Hydroxybenzoic acid ; 3.347: -ESI MRM Frag=100.0V CID@10.0 (137.0 -> 93.0) 20211020_uz2.d
3.347
Cpd 6: Chlorogenic acid ; 3.873: -ESI MRM Frag=100.0V CID@10.0 (353.0 -> 191.0) 20211020_uz2.d
3.873
4.337
Cpd 7: Syringic acid; 4.299: -ESI MRM Frag=100.0V CID@7.0 (197.0 -> 182.0) 20211020_uz2.d Smooth
4.299
Cpd 9: p-coumaric acid; 4.807: -ESI MRM Frag=100.0V CID@9.0 (163.0 -> 119.0) 20211020_uz2.d
4.807
4.343
5.022
Cpd 11: Vanillic acid; 4.856: -ESI MRM Frag=100.0V CID@15.0 (167.0 -> 108.0) 20211020_uz2.d
4.856
4.043
Cpd 13: Ferulic acid ; 5.079: -ESI MRM Frag=100.0V CID@11.0 (193.0 -> 134.0) 20211020_uz2.d
5.079
5.776
Cpd 14:Vanillin 5.687: -ESI MRM Frag=100.0V CID@21.0 (151.1 -> 92.0) 20211020_uz2.d
5.687
Cpd 14:Vanillin 5.687: -ESI MRM Frag=100.0V CID@17.0 (151.1 -> 136.0) 20211020_uz2.d
5.686
4.717
Counts (%) vs. Acquisition Time (min)

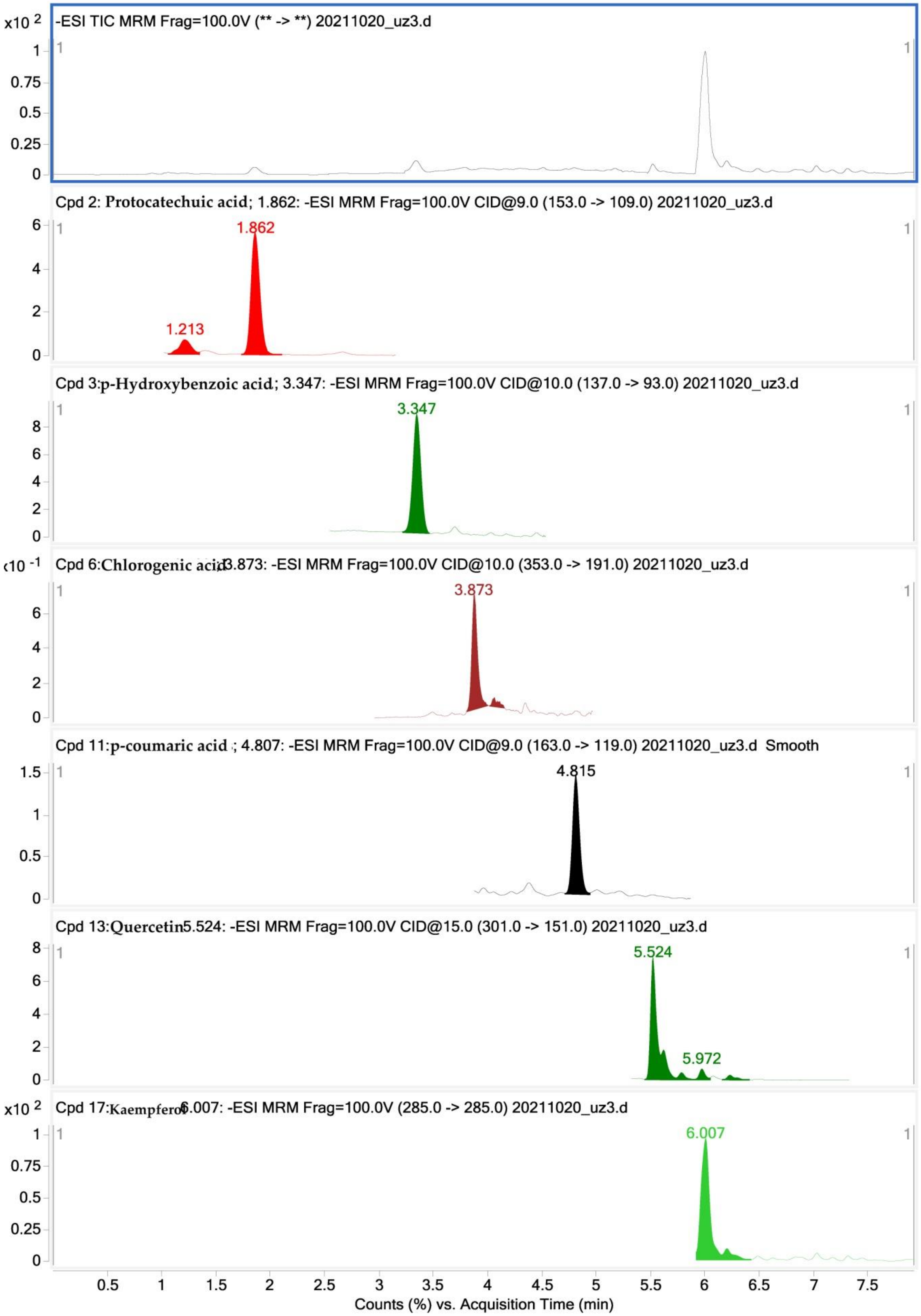

-ESI TIC MRM Frag=100.0V (** -> **) 20211020_uz3.d
Cpd 2: Protocatechuic acid; 1.862: -ESI MRM Frag=100.0V CID@9.0 (153.0 -> 109.0) 20211020_uz3.d
1.862
1.213
Cpd 3:p-Hydroxybenzoic acid; 3.347: -ESI MRM Frag=100.0V CID@10.0 (137.0 -> 93.0) 20211020_uz3.d
3.347
Cpd 6:Chlorogenic acid3.873: -ESI MRM Frag=100.0V CID@10.0 (353.0 -> 191.0) 20211020_uz3.d
3.873
Cpd 11:p-coumaric acid ; 4.807: -ESI MRM Frag=100.0V CID@9.0 (163.0 -> 119.0) 20211020_uz3.d Smooth
4.815
Cpd 13:Quercetin5.524: -ESI MRM Frag=100.0V CID@15.0 (301.0 -> 151.0) 20211020_uz3.d
5.524
5.972
Cpd 17:Kaempferol6.007: -ESI MRM Frag=100.0V (285.0 -> 285.0) 20211020_uz3.d
6.007
Counts (%) vs. Acquisition Time (min)

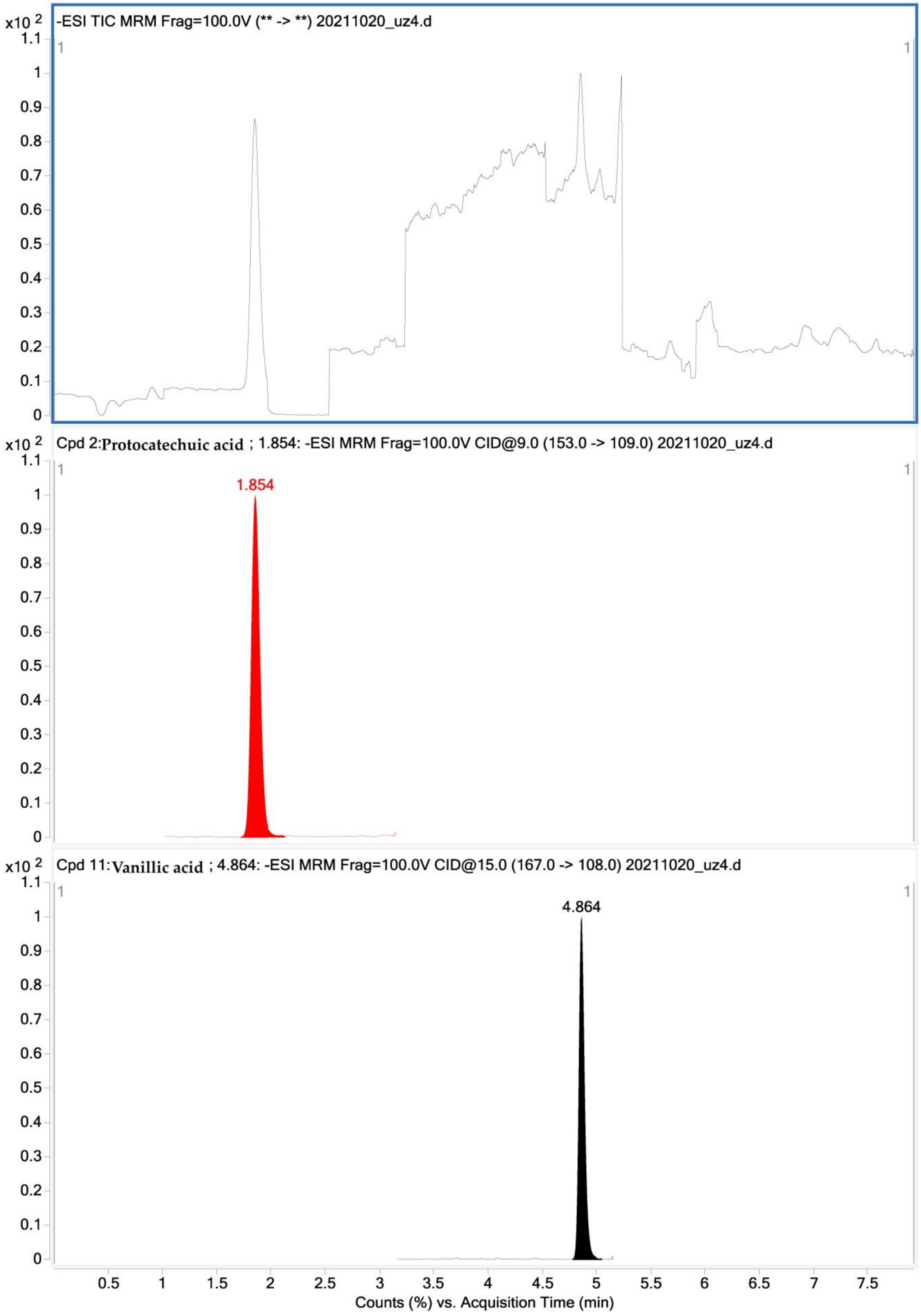

x10 2
-ESI TIC MRM Frag=100.0V (** -> **) 20211020_uz4.d
Cpd 2: Protocatechuic acid ; 1.854: -ESI MRM Frag=100.0V CID@9.0 (153.0 -> 109.0) 20211020_uz4.d
1.854
Cpd 11: Vanillic acid ; 4.864: -ESI MRM Frag=100.0V CID@15.0 (167.0 -> 108.0) 20211020_uz4.d
4.864
Counts (%) vs. Acquisition Time (min)

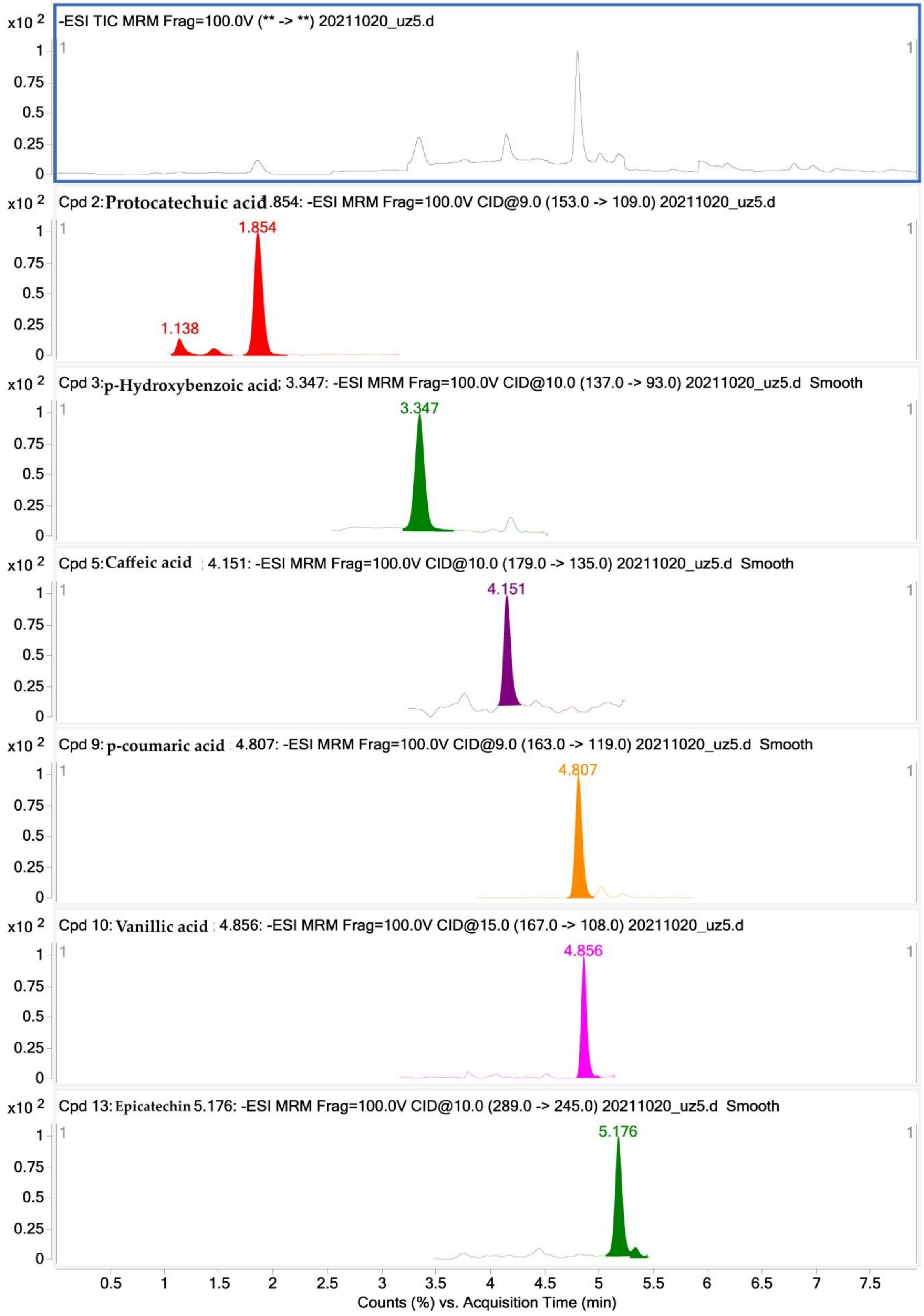

x10 2
-ESI TIC MRM Frag=100.0V (** -> **) 20211020_uz5.d
Cpd 2: Protocatechuic acid 1.854: -ESI MRM Frag=100.0V CID@9.0 (153.0 -> 109.0) 20211020_uz5.d
1.854
1.138
Cpd 3: p-Hydroxybenzoic acid 3.347: -ESI MRM Frag=100.0V CID@10.0 (137.0 -> 93.0) 20211020_uz5.d Smooth
3.347
Cpd 5: Caffeic acid 4.151: -ESI MRM Frag=100.0V CID@10.0 (179.0 -> 135.0) 20211020_uz5.d Smooth
4.151
Cpd 9: p-coumaric acid 4.807: -ESI MRM Frag=100.0V CID@9.0 (163.0 -> 119.0) 20211020_uz5.d Smooth
4.807
Cpd 10: Vanillic acid 4.856: -ESI MRM Frag=100.0V CID@15.0 (167.0 -> 108.0) 20211020_uz5.d
4.856
Cpd 13: Epicatechin 5.176: -ESI MRM Frag=100.0V CID@10.0 (289.0 -> 245.0) 20211020_uz5.d Smooth
5.176
Counts (%) vs. Acquisition Time (min)

Supplementary Materials: The following supporting information can be downloaded at: https://www.mdpi.com/article/10.3390/molecules29020000/s1, Table S1: Relationship between EC_{50} values in antioxidant activities and analyzed secondary metabolite content; Table S2: Relationship between IC_{50} values in enzyme inhibition and analyzed secondary metabolite content; Table S3: Selectivity of methanol extracts in antitumor action; Table S4: Mushroom species collected with corresponding family, habitat, sampling locations and usability. According to the map of Figure 1.; Figure S1: Total chromatograms (TIC) of all samples and one calibration sample (*F. pinicola*-uz 1, *F.betulina*-uz 2, *G. applanatum*-uz 3, *G. lucidum*-uz 4, *C. versicolor* –uz 5); Figure S2: Multiple reaction monitoring (MRM) transitions of polyphenols in analyzed samples (*F. pinicola* –uz 1, *F. betulina*-uz 2, *G. applanatum*-uz 3, *G. lucidum*-uz 4, *C. versicolor* –uz 5)

Author Contributions: Conceptualization, J.V., A.K. and M.K.; Methodology, J.V., A.K., M.K., B.Š-T. and Ž.Ž.; Software and Validation, S.M. and M.K.; Formal analysis, J.V., M.K., A.K., B.Š-T., Ž.Ž. and V.L.; Investigation, M.K., J.V. and A.K.; Resources, A.K., M.K. and J.V.; Data curation, M.K. and S.M.; writing – original draft preparation, M.K., J.V., SB.Š-T. and S.M.; writing – review and editing, M.K., J.V and S.M.; Visualization, M.K. and S.M.; Supervision, J.V. and A.K.; Project administration, A.K.; Funding acquisition, A.K., M.K. and J.V. All authors have read and agreed to the published version of the manuscript.

Funding: This research has been financially supported by the Ministry of Science, Technological Development and Innovation of Republic of Serbia (Contracts No: 451-03-47/2023-01/200051 and 451-03-47/2023-01/200026).

Acknowledgments: The authors are grateful to Snežana Spasić (Principal Research Fellow at the Institute of Chemistry, Technology and Metallurgy, University of Belgrade,) for the valuable comments and help during the preparation of original draft.

Conflicts of Interest: The authors declare no conflict of interest.
